# Morpholine-mediated defluorinative cycloaddition of *gem*-difluoroalkenes and organic azides

**DOI:** 10.3762/bjoc.19.111

**Published:** 2023-10-05

**Authors:** Tzu-Yu Huang, Mario Djugovski, Sweta Adhikari, Destinee L Manning, Sudeshna Roy

**Affiliations:** 1 Department of BioMolecular Sciences, School of Pharmacy, University of Mississippi, University, MS 38677, USA

**Keywords:** [3 + 2] cycloaddition, defluorination, fully decorated 1,2,3-triazoles, *gem-*difluoroalkenes, organic azides

## Abstract

Here, we report the first transition-metal-free defluorinative cycloaddition of *gem*-difluoroalkenes with organic azides in morpholine as a solvent to construct fully decorated morpholine-substituted 1,2,3-triazoles. Mechanistic studies revealed the formation of an addition–elimination intermediate of morpholine and *gem*-difluoroalkenes prior to the triazolization reaction via two plausible pathways. Attractive elements include the regioselective and straightforward direct synthesis of fully substituted 1,2,3-triazoles, which are otherwise difficult to access, from readily available starting materials.

## Introduction

**Figure 1:**
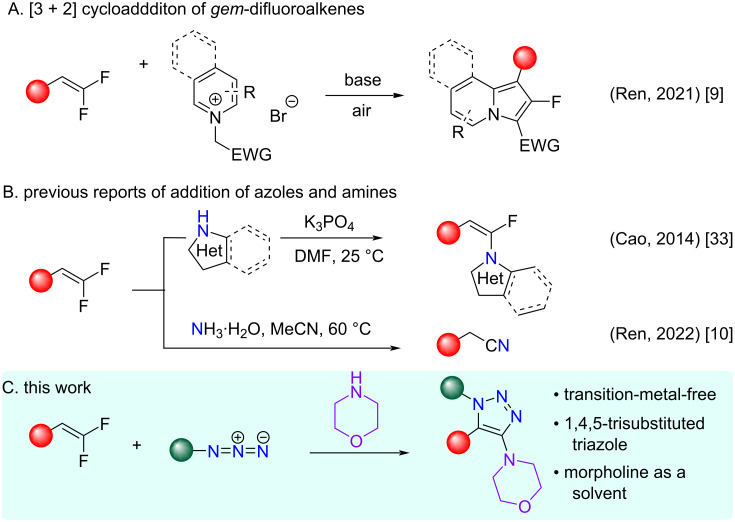
Functionalization of *gem-*difluoroalkenes with 1,3-dipoles and *N*-nucleophiles.

*gem*-Difluoroalkenes and their synthetic preparations soared in the last decade, driven by the high demand for carbonyl mimics in medicinal chemistry and drug discovery [1]. Although a wide array of functionalization strategies for *gem*-difluoroalkenes are available [2–3], only a couple of cycloaddition reactions has been reported [4]. For example, [3 + 2] dipolar cycloadditions to form saturated difluoroisoxazolidines [5–6] and difluoropyrrolidines [7] and [4 + 2] cycloaddition reactions with *gem-*difluoro-1,3-dienes [8]. The overall landscape of cycloaddition or addition–elimination reactions with 1,3-dipoles and *gem*-difluoroalkenes is largely unexplored and the only report of a cycloaddition is with 2-fluoroindolizines (Figure 1A) via a β-fluoride elimination in an S_N_V (nucleophilic vinylic substitution)-like transformation [9]. Nucleophilic addition reactions with azoles and amines (Figure 1B) are also well-precedented [10]. Herein, we address a critical gap in the literature and report the discovery of a cycloaddition of *gem*-difluoroalkenes and organic azides mediated by a base and with morpholine as a solvent. The cycloaddition adducts, 1,4,5-trisubstituted-1,2,3-triazoles, with a pendant morpholine at the C-4 position are formed with complete regiocontrol via β-fluoride elimination in an S_N_V-like transformation (Figure 1C).

1,2,3-Triazoles are a privileged scaffold in medicinal chemistry with a myriad of pharmacological activities against cancer [11–12], inflammation [13], bacterial [14–15], and viral infections [16]. Hence, new ways to rapidly and efficiently access 1,2,3-triazole heterocyclic motifs are still in demand. However, methods for the direct synthesis of 1,4,5-trisubstituted-1,2,3-triazoles are limited [17]. This is highly desirable since the selective introduction of substituents at three different positions on the 1,2,3-triazole ring can augment the features of the molecule. Triazoles are also found in many biologically important molecules and functionalized materials [11–16]. 1,4,5-Trisubstituted-1,2,3-triazoles are typically accessed in two ways: (1) direct synthesis using metal or metal-free catalysis and (2) post-functionalization of disubstituted-1,2,3-triazoles [17–18]. The direct synthesis of fully substituted triazoles entails either metal-free carbonyl-based [19–21] or metal-mediated and strain-promoted [22] azide–alkyne cycloaddition reactions [17,23–24]; however, most of these strategies use high temperatures [21,25]. Herein, we report the discovery of a novel, one-step regioselective method under mild conditions to obtain 1,4,5-trisubstituted-1,2,3-triazoles from *gem*-difluoroalkenes, organic azides, and morpholine.

Terminal *gem*-difluoroalkenes exhibit unique reactivity toward nucleophiles. The two σ-withdrawing fluorine atoms at the α-position and the strong polar nature of the double bond make *gem*-difluoroalkenes susceptible to a nucleophilic attack that is followed by a β-fluoride elimination, resulting in an S_N_V-like transformation [26]. We previously reported that α-fluoronitroalkenes could be effectively used as surrogates of α-fluoroalkynes in cycloaddition reactions with organic azides to construct 4-fluoro-1,5-disubstituted 1,2,3-triazoles regioselectively [27]. This two-step process involves an attack of the organic azide nucleophile to the β-position of α-fluoronitroalkenes. The polarity of *gem*-difluoroalkenes is reversed in comparison to α-fluoronitroalkenes since the nucleophile attacks at the α-position of the *gem*-difluoroalkenes. A cycloaddition reaction between organic azides and *gem*-difluoroalkenes in the presence of morpholine generates 1,5-disubstituted-1,2,3-triazoles with a pendant C-4 morpholine moiety. The regioselectivity of the triazole formation is dictated by morpholine preferentially making the first nucleophilic attack over azide at the α-position of *gem*-difluoroalkenes that subsequently undergoes a cycloaddition reaction.

## Results and Discussion

While investigating 1,3-dipolar cycloaddition reactions between organic azides and *gem*-difluoroalkenes to obtain the 4-fluoro-1,4-disubstituted 1,2,3-triazole regioisomers, we observed an interesting reactivity while screening different bases. In our optimization, we discovered, when morpholine was used in excess as a base, it generated fully substituted 1,2,3-triazole cycloaddition products with morpholine at the C-4 position instead of forming 5-fluorotriazoles. The fully substituted 1,2,3-triazoles are typically generated via an azide–alkyne cycloaddition or a multicomponent reaction between carbonyls and azides [17]. α-Trifluoromethyl (α-CF_3_) carbonyls were recently utilized to generate NH-1,2,3-triazoles and fully substituted 1,2,3-triazoles [28–29]. However, there are no reports of a formal [3 + 2] cycloaddition reaction utilizing *gem*-difluoroalkenes, which inherently exhibit attenuated activity compared to the activated α-CF_3_ carbonyls. This report provides a highly regioselective and novel way to access C-4-morpholine-functionalized fully decorated 1,2,3-triazoles from *gem*-difluoroalkenes and organic azides without the requirement of alkynes or late-stage modifications.

Our initial investigations led us to identify that adding morpholine as a solvent (0.34–0.4 M) in a reaction with 1-(2,2-difluoroethenyl)-4-methylbenzene (1 equiv) and phenyl azide (1.5 equiv) results in the formation of morpholine-substituted triazole **3’a** (entry 1, Table 1), in 21% yield, using NiCl_2_(PCy_3_)_2_ as a catalyst and K_3_PO_4_ as a base. A methyl handle on the *gem*-difluoroalkene **1** was used to aid in ^1^H NMR analysis. The *gem*-difluoroalkenes were synthesized in one step using sodium 2-chloro-2,2-difluoroacetate and triphenylphosphine in DMF at 100 °C for 5 h [30].

**Table 1:** Optimization of reaction conditions.^a^

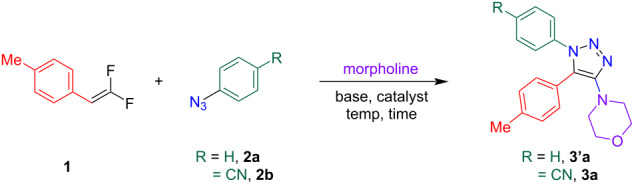						

entry	R	catalyst^b^ + additive (equiv)	base (equiv)	*T* (°C)	*t* (h)	yield (%)^c^

1	H^d^	NiCl_2_(PCy_3_)_2_	K_3_PO_4_ (2)	110	48	21
2	CN	NiCl_2_(PCy_3_)_2_	K_3_PO_4_ (2)	110	48	30
3	CN	NiCl_2_(dppp)_2_	K_3_PO_4_ (2)	110	48	54
4	CN	NiCl_2_(dppp)_2_	K_3_PO_4_ (2)	110	24	26
5	CN	NiCl_2_(dppp)_2_ + TMSCl (1)	K_3_PO_4_ (2)	110	24	11
6	CN	Cu(OAc)_2_	K_3_PO_4_ (2)	110	48	14
7	CN	CuCl (0.15)	K_3_PO_4_ (2)	110	48	11
8	CN^e^	NiCl_2_(dppp)_2_	NaH (1.2)	50	24	53
9	CN^e^	NiCl_2_(dppp)_2_	Cs_2_CO_3_ (2)	50	24	61
10	CN^e^	NiCl_2_(dppp)_2_	LiHMDS (0.4)	50	24	61
11	CN	NiCl_2_(dppp)_2_	LiHMDS (1)	50	24	28
12	CN	–	LiHMDS (0.4)	50	48	31
**13**	**CN**	**–**	**LiHMDS (0.4)**	**75**	**48**	**70**
14	CN	–	LiHMDS (0.2)	75	48	49
15	CN	–	LiHMDS (0.7)	75	48	41
16	CN	–	LiHMDS (1)	75	48	36
17	CN	–	Cs_2_CO_3_ (2)	75	48	61
18	CN^d^	–	K_3_PO_4_ (2)	110	48	57
19	H^d^	–	K_3_PO_4_ (2)	110	48	42
20	CN	–	LiHMDS (0.4)	75	24	36
21	CN	–	–	75	48	20

^a^Standard reaction conditions: 1 equiv of *gem*-difluoroalkene **1** (0.14 mmol), 1.5 equiv of aryl azide **2a** or **2b** (0.21 mmol) 0.4 equiv of LiHMDS (1 M in THF), and 0.3 mL morpholine (0.4 M) were mixed and heated at 75 °C. Changes in the molarity of morpholine did not affect the yield; ^b^0.1 equiv of catalyst used unless otherwise noted; ^c^isolated yield; ^d^2 equiv of azides, **2a** or **2b** were used; ^e^azide was added in two portions: first portion at *t* = 0 min and second portion at *t* = 6 h. For azide safety, please refer to Supporting Information File 1. The LiHMDS reagent was acquired from Thermo Scientific Chemicals as a 1 M solution in THF.

We hypothesized that electron-withdrawing *p*-cyanophenyl azide **2b**, would be better suited for optimizing the reaction conditions compared to the unsubstituted phenyl azide **2a**. Taking a clue from the literature, we looked at transition metals that facilitate defluorinative processes in *gem*-difluoroalkenes. NiCl_2_(PCy_3_)_2_ and NiCl_2_(dppp)_2_ were chosen for our initial investigations since they have been used in both the defluorination of *gem*-difluoroalkenes and the coordination with the azides to promote [3 + 2] cycloaddition reactions [2,31–32]. Based on our hypothesis, we observed that *p*-cyanophenyl azide (**2b**) gave a better yield (30%, Table 1, entry 2) compared to the unsubstituted phenyl azide (**2a**, 21% yield, entry 1). Among the nickel catalysts screened, NiCl_2_(dppp)_2_ gave a better yield (Table 1, entry 2 vs entry 3). K_3_PO_4_ was used as a base since it has been reported to facilitate the addition of azoles to *gem*-difluoroalkenes (Figure 1B) [9,33]. An elevated temperature (110 °C) was required along with 48 h reaction time (Table 1, entry 3 vs entry 4) due to the sluggish nature of the reaction and poor reactivity of the *gem*-difluoroalkenes. The decomposition of azides at higher temperatures required the use of **2a** or **2b** in excess. No significant difference in yields between 1.5 equiv and 2 equiv of the aryl azide was observed.

Adding fluorophilic additives (TMSCl, Table 1, entry 5) or using copper as other transition metal (CuCl or Cu(OAc)_2_, Table 1, entries 6 and 7) resulted in poor yields. Since the *gem*-difluoroalkenes are volatile compounds and as we observed decomposition of the azides at high temperatures resulting in reduced yields, we wanted to monitor the temperature and time course of this reaction. The time course study was carried out via ^19^F NMR spectroscopy to monitor the consumption of the *gem*-difluoro starting material **1**, which was completely consumed within 16 h (Figure 3). However, a 48 h time course gave a superior yield (Table 1, entry 13 vs entry 20). We hypothesize this might be due to the volatile nature of the *gem*-difluoroalkene and its existence in the vapor phase over the course of the reaction to facilitate reaction with the remainder of the azide. With the information on the temperature and time in hand, we next screened different bases (NaH, Cs_2_CO_3_, and LiHMDS) with the NiCl_2_(dppp)_2_ catalyst, which resulted in similar or improved yields up to 61% (Table 1, entries 8–10). We accidentally added 0.4 equiv of LiHMDS (1 M in THF) in the screening, which afforded the product with 61% yield (Table 1, entry 10). When 1 equiv of LiHMDS was used under otherwise identical conditions, we observed a lower yield of 28% (Table 1, entry 11). To determine the role of the catalyst, we next ran the reaction without catalyst using 0.4 equiv of LiHMDS at 50 °C, which afforded the product in 31% yield (Table 1, entry 12). In order to ascertain whether a higher temperature would improve the yield, we increased the temperature of the reaction to 75 °C, which afforded the best results (70%, Table 1, entry 13). When 0.2 equiv, 0.7 equiv, and 1 equiv of LiHMDS was used, a lower product yield of 58%, 50%, and 36%, respectively, was observed (Table 1, entries 14–16). This was surprising because there was no correlation between the amount of LiHMDS used versus the yields of the product formed.

Other bases, such as Cs_2_CO_3_ or K_3_PO_4_, resulted in slightly lower yields (Table 1, entries 17–19). Without any base or catalyst, the reaction yield was much lower (20%, Table 1, entry 21). A further screen of the concentration of the solvent (morpholine) or molarity of the reaction did not improve the yield (same or within 5%, see Supporting Information File 1, Table S1). We believe that LiHMDS gave the best results primarily because it is more miscible, resulting in a homogenous reaction mixture. LiHMDS being a strong base (p*K*_a_ ≈ 25.8) [34], facilitates the direct deprotonation of morpholine as opposed to acting as a scavenger base. Due to the significant difference in p*K*_a_ values between the conjugate acids of morpholine (p*K*_a_ of the conjugate acid is 8.3) [35] and LiHMDS, we posit that LiHMDS directly deprotonates morpholine. However, we cannot rule out that morpholine is acting as a scavenger base since it is used in large excess (0.4 M, which is equal to 30 equiv) compared to 0.4 equiv of LiHMDS and would buffer LiHMDS. Inorganic solid bases gave slightly decreased yields compared to LiHMDS (Table 1, entries 17–19 vs entry 13). Among the liquid bases that were screened, *N*,*N*-diisopropylethylamine (p*K*_a_ ≈ 9) gave the product in 38% yield, whereas NaHMDS afforded a 24% yield. Since LiHMDS gave the best yield thus far, we wanted to examine if Li^+^ ions play a role in the reaction. When the reaction was carried out with a different Li^+^ source (LiCl, 0.1 equiv) with a weaker base (Cs_2_CO_3_, p*K*_a_ of the conjugate acid 10.3) [36], it afforded the product in 29% yield, which is much poorer than under the previously optimized conditions (see Supporting Information File 1, Table S1). This observation suggests that Li^+^ ions act as a bystander and do not play a role in the reaction.

The reaction under the optimized conditions resulted in the formation of 4-(4-morpholino-5-(*p*-tolyl)-1*H*-1,2,3-triazole-1-yl)benzonitrile (**3a**) in 70% yield from 1 equiv of 1-(2,2-difluorovinyl)-4-methylbenzene and 1.5 equiv of 4-azidobenzonitrile with morpholine as solvent (0.4 M) and 0.4 equiv LiHMDS as a base at 75 °C for 48 h. The only byproducts observed are anilines as a result of thermal decomposition of the organic azides via reactive nitrene species. No other byproducts were observed by TLC or crude ^1^H NMR. The volatility of the *gem-*difluoroalkenes and the co-elution of the aniline byproducts during column chromatography with the desired products affected the overall yield of the reaction. For a complete optimization list with all conditions that were screened, see Supporting Information File 1.

**Figure 2:**
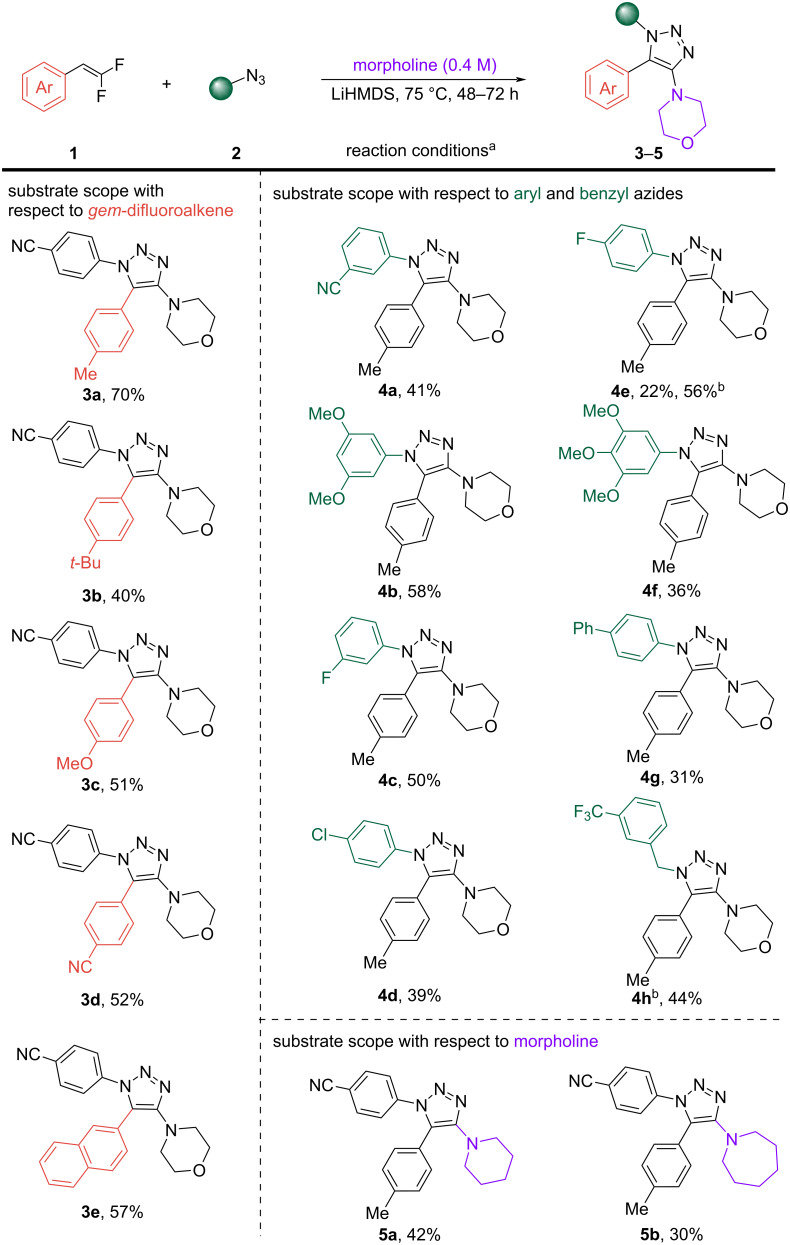
Substrate scope. Reaction conditions: **1** (1 equiv), **2** (1.5 equiv) 0.4 equiv of LiHMDS (1 M in THF), morpholine (0.34–0.4 M), 75 °C, 48 h. Isolated yields are reported. ^a^1 Equiv of CuSO_4_ was used as an additive_._^b^Modified reaction conditions for benzyl azides: **1** (1 equiv), **2** (1.5 equiv) 0.4 equiv of LiHMDS (1 M in THF), morpholine (0.34–0.4 M), 110 °C, 72 h.

With the optimized conditions in hand, we started exploring the substrate scope around the *gem*-difluoroalkene handle. As shown in Figure 2, electron-donating groups in the *para*-position, for instance, methyl (**3a**), *tert*-butyl (**3b**), and methoxy (**3c**) were tolerated affording the products in 40–70% yields. Also electron-withdrawing groups, such as cyano (**3d**) at the *para*-position, were amenable to the reaction conditions affording the product in 52% yield. Bulky groups, such as naphthalene were also suitable forming product **3e** in 57% yield, highlighting the functional group tolerability of this reaction.

Next, the scope of the reaction for aryl and benzyl azides was examined. An array of *para*- and *meta*-substituted aryl azides was amenable to the optimized conditions. The presence of electron-withdrawing groups worked well affording the products with *m*-cyano (**4a**), 3,5-dimethoxy (**4b**), *m*-fluoro (**4c**), and *p*-chloro (**4d**) substitution in 39–58% yields. It has to be noted, that CuSO_4_ (1 equiv) was used as an additive for the synthesis of product **4e** containing a *p*-fluoro substituent which improved the yield to 56%. Under regular optimized conditions without CuSO_4_, product **4e** was formed in only 22% yield. However, CuSO_4_ or any other Cu additives did not improve the yields when a cyano group was present on the azide handle. In fact, the use of CuSO_4_ with the cyano group lowered the yield (31%, see entry 12 in Table 1) which might be due to a coordination of the copper catalyst with the cyano group hindering the triazole formation [37]. The product **4f** containing a 3,4,5-trimethoxyphenyl substituent was afforded in a moderate 36% yield.

Electron-donating groups on the aryl azide, such as biphenyl at the *para*-position gave product **4g** in 31% yield. A clear trend was observed: electron-withdrawing groups on the aryl azides facilitated the reaction faster than electron-donating groups. Similar trends were observed for benzyl azides; however, this substituent was much less reactive compared to its aryl counterparts. It required a higher temperature of 110 °C and a longer duration of the reaction (72 h). The product with an electron-withdrawing group, such as trifluoromethyl (**4h**), was obtained in 44% yield. When morpholine was replaced with piperidine (**5a**) or seven-membered azepane (**5b**) as a solvent, a decreased yield was observed (30–42%). The addition of piperidine offers an advantage in expanding the substrate scope to medicinal chemistry applications. In the reaction with piperidine, we observed unreacted organic azide **2b** by TLC and ^1^H NMR analyses. Based on the ^1^H NMR analysis, 0.4 equiv of **2b** had reacted to form the product, 0.9 equiv of **2b** had decomposed to form aniline, and the remaining 0.2 equiv of **2b** was unreacted. Additionally, 30% of the aniline byproduct was also isolated, which explains the modest yields of this reaction and the sluggish nature.

**Figure 3:**
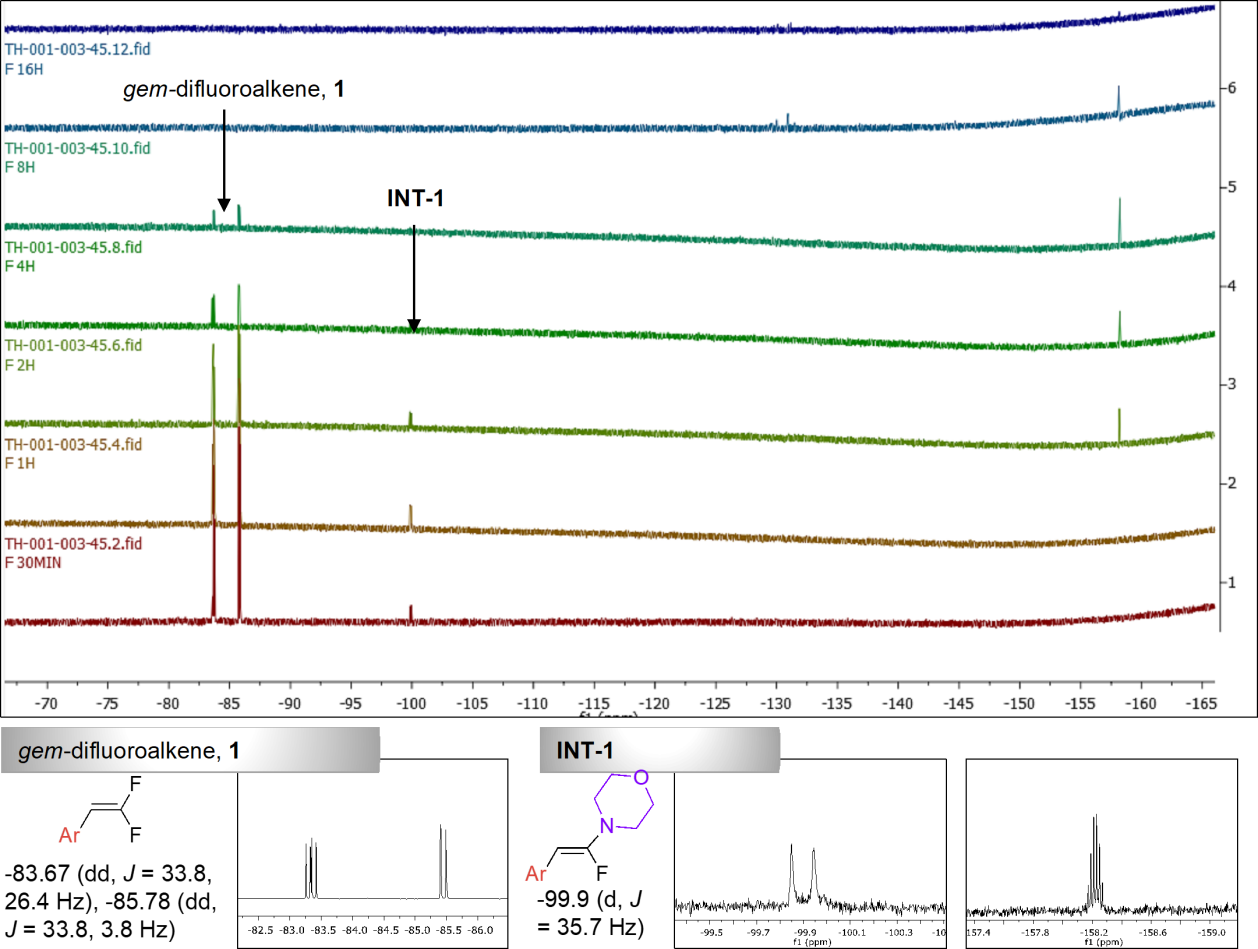
Time course profile monitored by ^19^F NMR spectroscopy.

**Figure 4:**
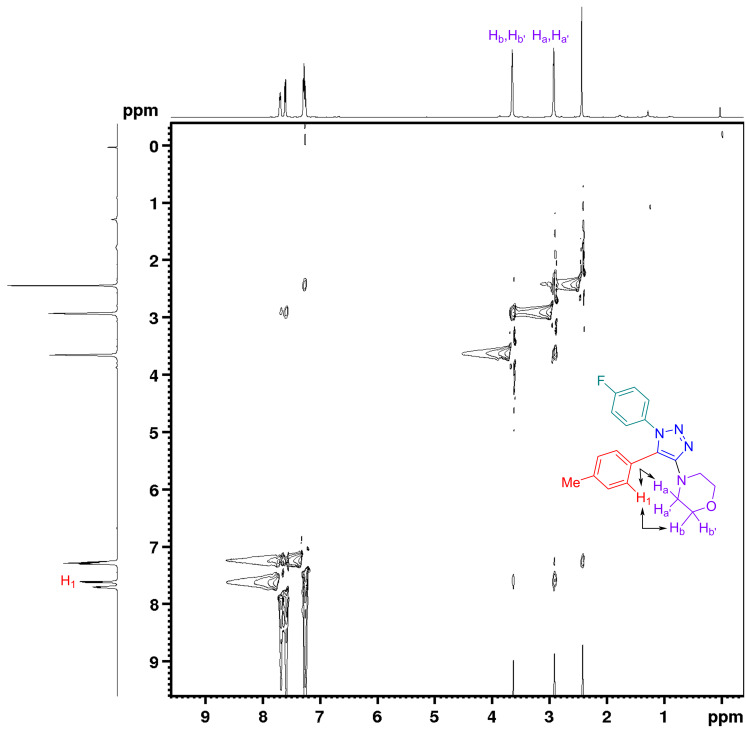
NOESY of **4e** confirming the regiochemistry of the product.

To investigate the mechanism of the current transformation, we conducted a series of experiments including a time course of the reaction using ^19^F NMR spectroscopy (Figure 3). We observed addition–elimination intermediate of morpholine and *gem*-difluoroalkenes INT-1, (−99.9 ppm, d, *J* = 35.7 Hz) within 30 min of the reaction and a gradual consumption of the *gem-*difluoroalkene **1** (−83.67 ppm, dd, *J* = 33.8, 26.4 Hz and −85.78, dd, *J* = 33.8, 3.8 Hz) throughout the course of 8 h and beyond. The *Z*-geometry of **INT-1** was determined from its ^3^*J*_H−F_ coupling constant of 35.7 Hz in the ^1^H NMR with a matching *J* value in the ^19^F NMR. This is in agreement with Cao’s report on the geometry of *N*-(α-fluorovinyl)azoles [33]. The configurations of the *E*- and *Z*-isomers were determined by their ^3^*J*_H−F_ coupling constants in the ^1^H NMR spectra, circa 32.0 Hz for *Z*-isomers and 8.0 Hz for *E*-isomers [33]. A peak was observed at −158.2 ppm in the ^19^F NMR spectrum after 2 h of the reaction, which could be the fluoride salt of the dimorpholine adduct. This peak was also found when the reaction was run in the absence of azide using optimized conditions (see Supporting Information File 1, mechanistic study, section 8). However, its further characterization was not possible because it disappeared upon workup. Finally, a 2D NOESY experiment was utilized to confirm the regiochemistry of 4-(1-(4-fluorophenyl)-5-(*p*-tolyl)-1*H*-1,2,3-triazol-4-yl)morpholine (**4e**), one of the fully decorated 1,2,3-triazoles (Figure 4). The peak at 7.59 ppm (d, *J* = 8.1 Hz) in the ^1^H NMR spectrum corresponding to the H_1_ protons of the C-5-aryl substituent on the 1,2,3-triazole ring shows a cross-peak with the protons of the C-4-morpholine unit (H_a_ = 3.68–3.59 ppm, m and H_b_ = 2.94–2.86 ppm, m). This suggests they are adjacent in space, thereby confirming the 1,5-disubstituted pattern on the 1,2,3-triazole ring with the morpholine moiety attached at the C-4 position. The distance between the H_1_ aryl proton and the morpholine protons was determined to be 2.3 Å (H_1_↔H_a_), 2.6 Å (H_1_↔H_a_′), and 4.5 Å (H_1_↔H_b_), 4.7 Å (H_1_↔H_b_′) (see Supporting Information File 1, regioisomer study, section 9, for more details).

**Figure 5:**
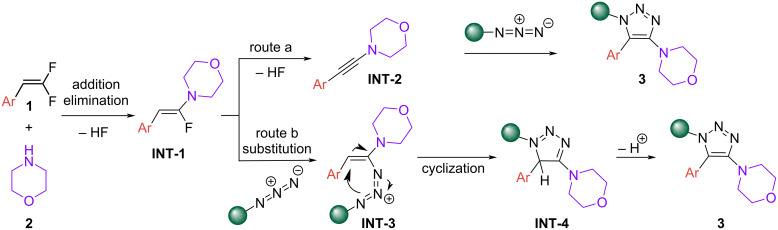
Proposed mechanism.

Based on these experiments and literature reports [28,33], we propose a base-mediated nucleophilic addition–elimination of morpholine to *gem*-difluoroalkene **1** affording **INT-1**, which can generate product **3** via two routes (Figure 5). Route A entails the formation of an aminoalkyne intermediate, **INT-2**, which can participate in a [3 + 2] azide–alkyne cycloaddition to form the final product **3**. Alternatively, vinylic azido amine intermediate **INT-3** can be formed via vinylic substitution of **INT-1** with an azide which can cyclize to form **INT-4** that subsequently aromatizes to afford product **3** (route B).

**Figure 6:**
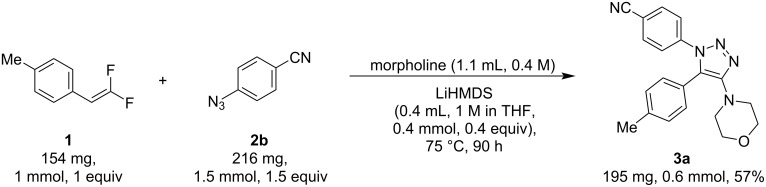
Scale-up experiment.

To demonstrate the applicability of this method, a scale-up reaction was performed using 150 mg of the limiting reagent, which is five times the usual reaction scale used in substrate scope screening or optimization experiments (Figure 6). In this scale-up experiment, we obtained the product with 57% yield , which is slightly lower than 70% using 1-(2,2-difluorovinyl)-4-methylbenzene (**1**, 154 mg, 1 mmol, 1 equiv), 4-azidobenzonitrile (**2b**, 216 mg, 1.5 mmol, 1.5 equiv), and LiHMDS (0.4 mL, 1 M in THF, 0.4 mmol, 0.4 equiv) in morpholine (1.1 mL, 0.4 M) at 75 °C. The 4-azidobenzonitrile (**2b**) was added in two portions of 0.75 equiv at *t* = 0 min and the remainder 0.75 equiv were added at *t* = 16 h. This addition strategy aimed to mitigate the decomposition of 4-azidobenzonitrile (**2b**) during the extended reaction duration. The progress of the reaction was monitored via TLC, and starting material **1** was still observed at 48 h. The reaction ran for a total of 90 h until all the starting materials were consumed and 195 mg (57%) of product **3a** was obtained. This shows the synthetic utility of this method; however, additional investigations into process chemistry may be necessary to accommodate a larger reaction scale.

## Conclusion

In conclusion, we have shown for the first time a [3 + 2] cycloaddition of *gem*-difluoroalkenes with organic azides in morpholine as a solvent forming C-4-morpholine functionalized fully decorated 1,2,3-triazoles with potential applications in pharmaceutical, biomedical, agrichemical, and materials sciences. This study fills a critical gap in the literature as it is a transition-metal-free and regioselective reaction that does not rely on carbonyl- or alkyne-based methods or late-stage modifications to access 1,4,5-trisubstituted-1,2,3-triazoles. However, carbonyl chemistry was utilized to synthesize the *gem*-difluoroalkene starting material [30]. In fact, our findings offer a straightforward direct synthesis of fully substituted 1,2,3-triazoles, which are otherwise difficult to access, from readily available starting materials. ^19^F NMR studies indicate a mechanism involving an addition–elimination intermediate of morpholine and *gem*-difluoroalkenes that subsequently undergoes a [3 + 2] cycloaddition with an organic azide. A relatively wide range of 1,4,5-trisubstituted-1,2,3-triazoles was obtained in 30–70% yields with high regioselectivity and modest functional group tolerability. This work demonstrates that *gem*-difluoroalkenes can serve as versatile fluorinated building blocks in lieu of alkynes to access a set of fully decorated 1,2,3-triazoles.

## Supporting Information

File 1 General information, experimental procedures for all the substrates and intermediates, characterization data, and NMR spectra (^1^H, ^19^F, and ^13^C NMR).
