# Supplementary material for: Morpholine-mediated defluorinative cycloaddition of gem-difluoroalkenes and organic azides
Source: Beilstein J Org Chem. 2023 Oct 5;19:1545–54. doi: 10.3762/bjoc.19.111 (PMC10562644; doi:10.3762/bjoc.19.111)
Supplement: File 1 — General information, experimental procedures for all the substrates and intermediates, characterization data, and NMR spectra (1H, 19F, and 13C NMR). [file Beilstein_J_Org_Chem-19-1545-s001.pdf]

BEILSTEIN JOURNAL OF ORGANIC CHEMISTRY

# Supporting Information

for

# Morpholine-mediated defluorinative cycloaddition of *gem*-difluoroalkenes and organic azides

Tzu-Yu Huang, Mario Djugovski, Sweta Adhikari, Destinee L. Manning and Sudeshna Roy

*Beilstein J. Org. Chem.* **2023,** *19,* 1545–1554. doi:10.3762/bjoc.19.111

# General information, experimental procedures for all the substrates and intermediates, characterization data, and NMR spectra ($^{1}H$, $^{19}F$, and $^{13}C$ NMR)

License and Terms: This is a supporting information file under the terms of the Creative Commons Attribution License (https://creativecommons.org/licenses/by/4.0). Please note that the reuse, redistribution and reproduction in particular requires that the author(s) and source are credited and that individual graphics may be subject to special legal provisions.
The license is subject to the *Beilstein Journal of Organic Chemistry* terms and conditions: (https://www.beilstein-journals.org/bjoc/terms)

**Table of contents**

1. General information.........S2

2. Azides safety.........S2

3. Optimization studies:.........S3

4. General procedure for synthesizing *gem*-difluoro olefins.........S6

5. General procedure for the synthesis of organic azides.........S8

Method A:.........S8

Method B:.........S8

6. General procedure for the synthesis of 1,4,5-trisubstituted-1,2,3-triazoles.........S12

7. Time course study.........S22

8. Mechanistic study.........S23

9. Regioisomer study:.........S26

10. Scale-up experiment:.........S28

11. $^{1}H$, $^{19}F$ and, $^{13}C$ NMR spectra:.........S29

12. References:.........S46

## 1. General information

Unless otherwise noted, all reactions were carried out under argon atmosphere. All commercially available reagents were used without further purification. All of the solvents were treated according to known methods. For TLC, Sorbtech silica XG TLC plates w/UV254 indicator was used and visualized under a UV lamp. Flash column chromatography was performed in Biotage Isolera One with Biotage SNAP 10–50g cartridges. $^{1}H$, $^{19}F$, and $^{13}C$ NMR spectra were recorded on a Bruker Avance-500 (125 MHz) spectrometer and chemical shifts are reported in ppm (δ) using deuterated solvents for $^{1}H$, $^{13}C$ NMR, and $^{19}F$ NMR. $CDCl_3$ (δ = 77.16 ppm) for $^{13}C$ NMR, $CFCl_3$ (δ = 0 ppm) for $^{19}F$ NMR, and $CDCl_3$ (δ = 7.26 ppm) for $^{1}H$ NMR were used as internal standards.  HRMS was recorded using quadruple-TOF was used to obtain the data both in positive and negative modes. ATR-IR was taken using an Agilent Technologies Cary 600series FTIR Spectrometer. Melting point was recorded using the Stanford Research System OptiMelt Automated Melting Point System. Data were reported as follows: chemical shift, multiplicity (s = singlet, d = doublet, t = triplet, q = quartet, dd = doublet of doublets, td = triplet of doublets, qd = quartet of doublets, h = m = multiplet), coupling constants (Hz) and integration.

LiHMDS was acquired from Thermo Scientific Chemicals in a 100 mL glass container, appropriately labeled as lithium bis(trimethylsilyl)amide, 1 M solution in THF/ethylbenzene, AcroSeal™, Thermo Scientific Chemicals. Its corresponding catalog number: 347701000.

## 2. Azides safety

Caution must always be exercised when working with azides. It is important to ensure that the carbon-to-nitrogen ratio in organic azides remains above three. An alternative guideline known as the "rule of six" dictates that there should be a minimum of six carbon atoms per energetic functional group. Organic azides, especially those with low molecular weight or high nitrogen content, have the potential to be explosive. The application of heat, light, or pressure can trigger the decomposition of azides. Additionally, the azide ion is toxic, necessitating the use of gloves when handling sodium azide. When conducting experiments involving heating azides in the presence of copper, a blast shield should always be employed. It is crucial to never mix azides or their waste with acidic, metallic, or halogenated solvents.

### 3. Optimization studies:

Table S1. Optimization of reaction conditions[a]

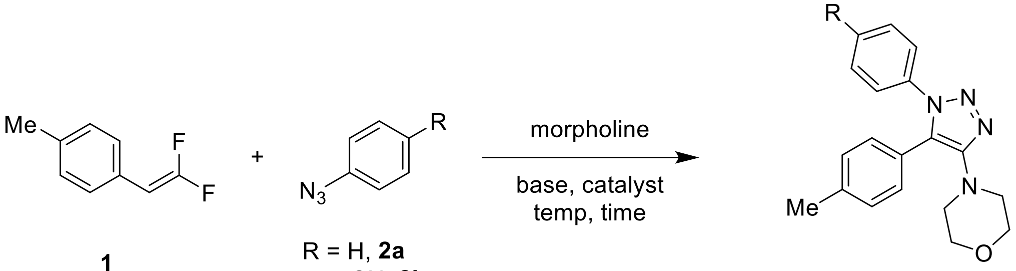


1

R = H, **2a**
= CN, **2b**
= F, **2c**

R = H, **3'a**
= CN, **3a**
= F, **3b**

| entry | R | Catalyst[c] | Base (equiv) | Solvent | T, °C | t, (h) | Yield %[d] |
|---|---|---|---|---|---|---|---|
| ***Molarity*** | | | | | | | |
| 1 | H[b] | - | $K_3PO_4$ (2) | Morpholine (0.7 M) | 110 | 48 | 39 |
| 2 | H[b] | - | $K_3PO_4$ (2) | Morpholine (0.5 M) | 110 | 48 | 42 |
| 3 | H[b] | - | $K_3PO_4$ (2) | Morpholine (0.2 M) | 110 | 48 | 30 |
| ***Temperature*** | | | | | | | |
| 4 | CN | $NiCl_2(dppp)_2$ | $K_3PO_4$ (2) | Morpholine (0.4 M) | 100 | 24 | 36 |
| 5 | CN | $NiCl_2(dppp)_2$ | $K_3PO_4$ (2) | Morpholine (0.4 M) | 75 | 24 | 44 |
| 6 | CN | $NiCl_2(dppp)_2$ | $K_3PO_4$ (2) | Morpholine (0.4 M) | 50 | 24 | 35 |
| 7 | CN | $NiCl_2(dppp)_2$ | LiHMDS (0.4) | Morpholine (0.4 M) | 75 | 48 | 40 |
| 8 | CN | $NiCl_2(dppp)_2$ | LiHMDS (0.4) | Morpholine (0.4 M) | 25 | 48 | 0 |
| 9 | CN | $NiCl_2(dppp)_2$ | LiHMDS (0.4) | Morpholine (0.4 M) | 0 | 48 | 0 |
| ***Base*** | | | | | | | |
| 10 | F | $CuSO_4$ (1) | DIPEA (2) | Morpholine (0.4 M) | 75 | 48 | 38[e] |
| 11 | F | $CuSO_4$ (1) | NaHMDS (2) | Morpholine (0.4 M) | 75 | 48 | 24[e] |
| ***Lithium Source*** | | | | | | | |
| 12 | CN | LiCl (0.1) | $Cs_2CO_3$ (2) | Morpholine (0.4 M) | 75 | 48 | 29 |
| ***Solvent*** | | | | | | | |
| 13 | H[b] | - | $K_3PO_4$ (2) | Morpholine (0.4 M), DMF (10 equiv) | 110 | 48 | 35 |
| 14 | H[b] | - | $K_3PO_4$ (2) | Morpholine (10 equiv), DMF (250 equiv) | 110 | 48 | 27 |
| 15 | CN | - | $K_3PO_4$ (2) | 1,4-Dioxane (0.3 M) | 95 | 48 | 0 |
| 16 | CN | - | LiHMDS (0.4) | 2-Pyrrolidinone (0.4 M) | 75 | 48 | 0 |
| 17 | CN | - | LiHMDS (0.4) | Morpholine (0.4 M), Toluene (20 equiv) | 75 | 48 | 36 |
| 18 | F | $CuSO_4$ (1) | $Cs_2CO_3$ (2) | Morpholine (20 equiv), DMF (0.4 M) | 75 | 48 | 53[e] |
| 19 | F | $CuSO_4$ (1) | $Cs_2CO_3$ (2) | Morpholine (10 equiv), DMF (0.4 M) | 75 | 48 | 37[e] |

| | | | | | | | |
|---|---|---|---|---|---|---|---|
| 20 | F | $CuSO_4$ (1) | $Cs_2CO_3$ (2) | Morpholine (5 equiv), DMF (0.4 M) | 75 | 48 | 38[e] |
| 21 | F | $CuSO_4$ (1) | $Cs_2CO_3$ (2) | Morpholine (2 equiv), DMF (0.4 M) | 75 | 48 | 25[e] |
| ***Microwave conditions*** | | | | | | | |
| 22 | CN | - | LiHMDS (0.4) | Morpholine (0.4 M) | 135 | 1.5 | 15 |
| ***Two-Portion Addition*** | | | | | | | |
| 23[f] | CN | - | LiHMDS (0.4) | Morpholine (0.4 M) | 75 | 48 | 15 |
| ***Scale up of reaction*** | | | | | | | |
| 24 | CN | - | LiHMDS (0.4) | Morpholine (0.4 M) | 75 | 90 | 57 |

[a]Standard reaction conditions: 1 equiv of *gem*-difluoroalkene **1** (0.14 mmol), 1.5 equiv of aryl azide **2a** and **2b** (0.21 mmol), and 0.3 mL morpholine (0.4 M) were mixed and heated at 110 °C. [b] 2.0 Equiv of azide was used. [c]0.1 Equiv of catalyst used unless otherwise noted. [d]Isolated yield. [e] *para*-Fluorophenyl azide was used for screening to facilitate reaction monitoring via $^{19}F$ NMR. Yield was obtained by utilizing the relative integration. [f] 1.5 equiv of *gem*-difluoroalkene **1** (0.14 mmol), and 1equiv of aryl azide **2b** (0.21 mmol) were used. The 2,2-difluorovinylarene **1** was added in two portions with 0.75 equiv at t = 0 min and the remainder 0.75 equiv was added at t = 16 h.

[e]The yield was obtained by utilizing the relative integration

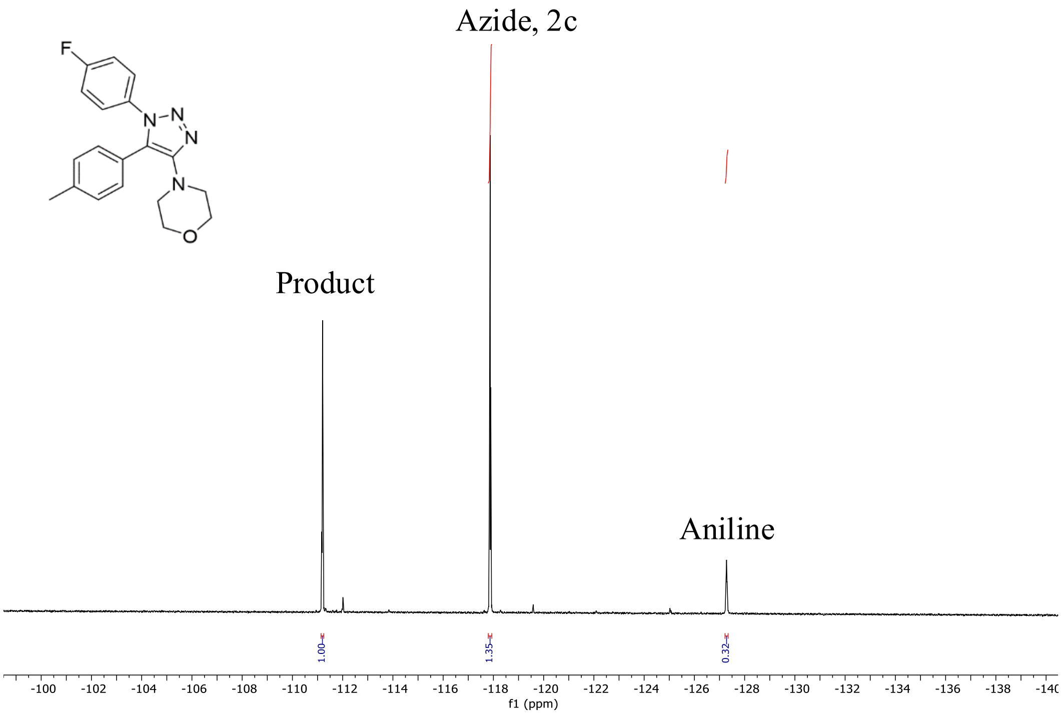


**Figure S1**. The yield was obtained by utilizing the relative integration.

Entry 18: 1.00/1.00+1.35+0.32=37%

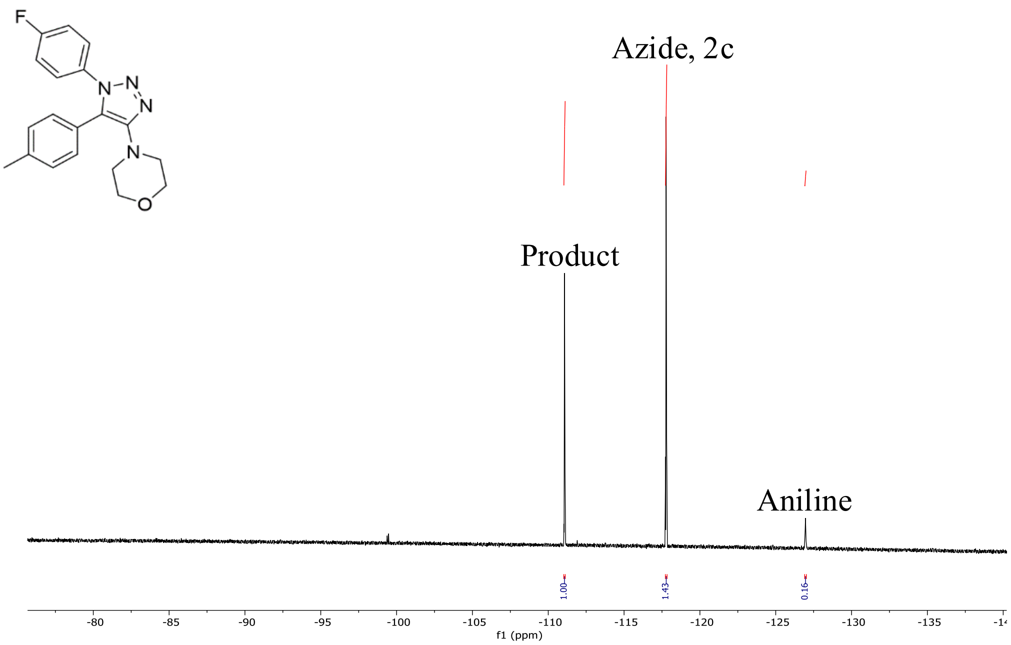


**Figure S2**. The yield was obtained by utilizing the relative integration.

Entry 19: 1.00/1.00+1.43+0.16=38%

## 4. General procedure for synthesizing *gem*-difluoro olefins

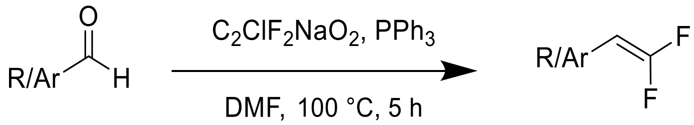


In an oven-dried, Ar-flushed vial charged with a stirring bar, sodium 2-chloro-2,2-difluoroacetate (6.0 mmol, 1.5 equiv) and triphenylphosphine (5.0 mmol, 1.2 equiv) were added. The vial was then vacuumed and flushed with Ar three times. Next, DMF (1.20 mL, 1.5 mL/mmol) was added and stirred until the reaction mixture became homogenous. Once mixed, the appropriate aldehyde (0.8 mmol, 1 equiv) was added and stirred at 100 °C under Ar. Caution when using balloons due to carbon dioxide evolution. The reaction mixture was stirred for 6 h or until completion (monitored by TLC or $^{19}$F NMR spectroscopy) under the same conditions.

### 1-(2,2-Difluorovinyl)-4-methylbenzene (1)

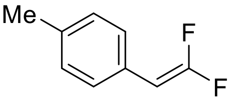


**1-(2,2-Difluorovinyl)-4-methylbenzene (1)** was prepared following the general procedure using 4-methylbenzaldehyde (500 mg, 4.2 mmol), sodium 2-chloro-2,2-difluoroacetate (951 mg, 6.2 mmol) and triphenylphosphine (1.3 g, 5.0 mmol). The product was obtained in 65% yield (418 mg) as a colorless oil.

**$^1$H NMR** (400 MHz, Chloroform-*d*) δ 7.23 (d, *J* = 8.2 Hz, 2H), 7.15 (d, *J* = 8.0 Hz, 2H), 5.21 (d, *J* = 3.9 Hz, 1H), 2.34 (s, 3H).
**$^{19}$F NMR** (377 MHz, Chloroform-*d*) δ -83.69 (dd, *J* = 33.8, 26.4 Hz), -85.81 (dd, *J* = 33.8, 3.8 Hz).
Spectroscopic data for **(1)** are consistent with previously reported data for this compound [1].

### 1-(*tert*-Butyl)-4-(2,2-difluorovinyl)benzene (1b)

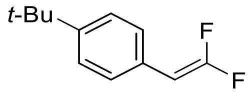


**1-(*tert*-Butyl)-4-(2,2-difluorovinyl)benzene (1b)** was prepared following the general procedure using benzaldehyde,4-(1,1-dimethylethyl (250 mg, 1.5 mmol), sodium 2-chloro-2,2-difluoroacetate (352 mg, 2.3 mmol) and triphenylphosphine (485 mg, 1.8 mmol). The product was obtained in 75% yield (228 mg) as a reddish-brown oil.

**$^1$H NMR** (400 MHz, Chloroform-*d*) δ 7.44–7.25 (m, 4H), 5.28 (dd, *J* = 26.5, 3.8 Hz, 1H), 1.35 (s, 9H).
**$^{19}$F NMR** (376 MHz, Chloroform-*d*) δ -83.59 (dd, *J* = 33.2, 26.3 Hz), -85.54 (dd, *J* = 33.4, 4.0 Hz).
Spectroscopic data for **(1b)** are consistent with previously reported data for this compound [2].

**1-(2,2-Difluorovinyl)-4-methoxybenzene (1c)**

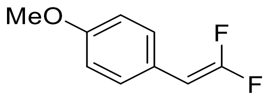


**1-(2,2-Difluorovinyl)-4-methoxybenzene (1c)** was prepared following the general procedure using 4-methoxybenzaldehyde (500 mg, 3.7 mmol), sodium 2-chloro-2,2-difluoroacetate (840 mg, 5.5 mmol) and triphenylphosphine (1.2 g, 4.4 mmol). The product was obtained in 80% yield (501 mg) as a colorless oil.
**$^1$H NMR** (400 MHz, Chloroform-*d*) δ 7.23 (d, *J* = 8.2 Hz, 2H), 7.15 (d, *J* = 8.0 Hz, 2H), 5.21 (d, *J* = 3.9 Hz, 1H), 2.34 (s, 3H).
**$^{19}$F NMR** (376 MHz, Chloroform-*d*) δ -85.18 (dd, *J* = 36.8, 26.5 Hz), -86.98 (dd, *J* = 36.8, 4.0 Hz).
Spectroscopic data for **(1c)** are consistent with previously reported data for this compound [3].

**1-(2,2-Difluoroethenyl)-4-benzonitrile (1d)**

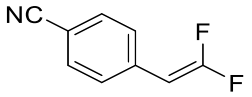


**1-(2,2-Difluoroethenyl)-4-benzonitrile (1d)** was prepared following the general procedure using 4-formylbenzonitrile (243 mg, 1.9 mmol), sodium 2-chloro-2,2-difluoroacetate (426mg, 2.8 mmol) and triphenylphosphine (586mg, 2.2 mmol). The product was obtained in 69% yield (212 mg) as a colorless oil.

**$^1$H NMR** (400 MHz, Chloroform-*d*) δ 7.62 (d, *J* = 8.5 Hz, 2H), 7.42 (d, *J* = 8.5 Hz, 2H), 5.34 (dd, *J* = 25.5, 3.4 Hz, 1H).
**$^{19}$F NMR** (377 MHz, Chloroform-*d*) δ -78.32 (dd, *J* = 25.4, 20.5 Hz), -79.98 (dd, *J* = 20.5, 3.4 Hz).
Spectroscopic data for **(1e)** are consistent with previously reported data for this compound [4].

**2-(2,2-Difluorovinyl)naphthalene (1e)**

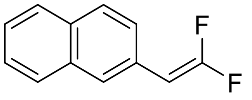


**2-(2,2-Difluorovinyl)naphthalene (1e)** was prepared following the general procedure using naphthalene-2-carbaldehyde (260 mg, 1.7 mmol), sodium 2-chloro-2,2-difluoroacetate (382 mg, 2.5 mmol) and triphenylphosphine (526 mg, 2.0 mmol). The product was obtained in 88% yield (278 mg) as a colorless oil**.**

**$^1$H NMR** (400 MHz, Chloroform-*d*) δ 7.84–7.73 (m, 4H), 7.52–7.42 (m, 3H), 5.44 (dd, *J* = 26.2, 3.8 Hz, 1H).
**$^{19}$F NMR** (377 MHz, Chloroform-*d*) δ -82.44 (dd, *J* = 30.7, 26.0 Hz), -84.16 (dd, *J* = 30.9, 3.8 Hz).
Spectroscopic data for **(1e)** are consistent with previously reported data for this compound [4].

## 5. General procedure for the synthesis of organic azides

**Method A:**

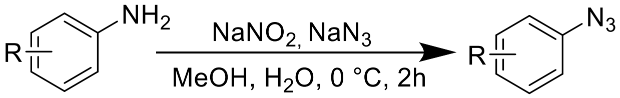


The corresponding aniline (500 mg, 1 equiv) was suspended in methanol (4 mL) and water (3 ml). Then, HCl (2 mL) was added at 0 °C to the mixture which was stirred for another 5 minutes. The mixture was stirred at 0 °C for 20 minutes after which sodium nitrite (1.2 equiv) in water (1.5 mL) was added dropwise over 10 minutes. After a solution of sodium azide (1.2 equiv) in water (1.5 mL) was added dropwise to the reaction mixture for over 10 minutes, the reaction mixture was stirred at room temperature for 2 h or until completion (monitored by TLC) under the same conditions. The reaction was quenched with water and the organic layer was extracted with ethyl acetate (× 3), dried with anhydrous $Na_2SO_4$, and concentrated using a rotavap. Unless other noted, the organic azides were used without further purification.

**Warning:** Working with azides demands caution due to potential explosiveness. This reaction can form hydrazoic acid which is highly explosive. Guidelines include a carbon-to-nitrogen ratio above three and the "rule of six," requiring six carbon atoms per energetic group. High-nitrogen organic azides are sensitive to heat, light, and pressure, possibly exploding. Sodium azide, with toxic azide ions, needs glove use. Copper-heated azides require a blast shield. Avoid mixing azides or waste with acidic, metallic, or halogenated solvents for safety. For more information, please see the warning on page 2 of the SI.

**Method B:**

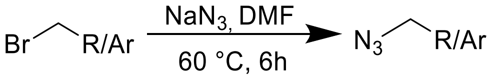


The corresponding aniline (500 mg, 1 equiv) and sodium azide (1.2 equiv) were suspended in DMF (2 mL, 1 M) and the reaction mixture was stirred at 60 °C for 6 h or until completion (monitored by TLC). The reaction was quenched with brine and the organic layer was extracted with ethyl acetate (× 3), dried with anhydrous $Na_2SO_4$, and concentrated in vacuo. The products were obtained after purification by column chromatography (gradient: 0–20% ethyl acetate in hexane) on silica gel.

### 4-Azidobenzonitrile (2)

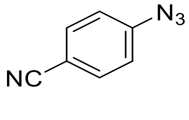


**4-Azidobenzonitrile (2)** was prepared following method A using 4-aminobenzonitrile (500 mg, 4.2 mmol), sodium nitrite (350 mg, 5.0 mmol), and sodium azide (330 mg, 5.0 mmol). The product was obtained in 89% yield (546 mg) as a yellow solid.

**$^1$H NMR** (400 MHz, Chloroform-*d*) δ 7.70–7.63 (m, 2H), 7.16–7.09 (m, 2H).
Spectroscopic data for **(2)** is consistent with previously reported data for this compound [5]**.**

**3-Azidobenzonitrile (2a)**

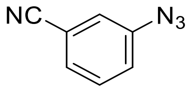


**3-Azidobenzonitrile (2a)** was prepared following method A using 3-aminobenzonitrile (257 mg, 2.1 mmol), sodium nitrite (180 mg, 2.6 mmol), and sodium azide (170 mg, 2.6 mmol). The product was obtained in 92% yield (287 mg) as a brown solid.

**$^1$H NMR** (400 MHz, Chloroform-d) δ 7.58 – 7.40 (m, 2H), 7.37 – 7.22 (m, 2H).
Spectroscopic data for **(2a)** is consistent with previously reported data for this compound [6].

**1-Azido-3,5-dimethoxybenzene (2b)**

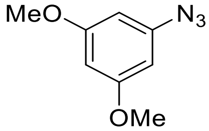


**1-Azido-3,5-dimethoxybenzene (2b)** was prepared following method A using 3,5-dimethoxyaniline (250 mg, 1.6 mmol), sodium nitrite (135 mg, 2.0 mmol), and sodium azide (127 mg, 2.0 mmol). The product was obtained in 86% yield (250 mg) as a brown solid after extraction.

**$^1$H NMR** (400 MHz, Chloroform-*d*) δ 6.22 (dd, *J* = 22.9, 2.2 Hz, 3H), 3.78 (s, 6H).
Spectroscopic data for **(2b)** is consistent with previously reported data for this compound [7].

**1-Azido-3-fluorobenzene (2c)**

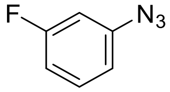


**1-Azido-3-fluorobenzene (2c)** was prepared following method A using 3-fluoroaniline (250 mg, 2.3 mmol), sodium nitrite (193 mg, 2.8 mmol), and sodium azide (182 mg, 2.8 mmol). The product was obtained in 41% yield (128 mg) as a yellow liquid after extraction.

**$^1$H NMR** (400 MHz, Chloroform-*d*) δ 7.31 (td, *J* = 8.2, 6.2 Hz, 1H), 6.89–6.81 (m, 2H), 6.74 (dt, *J* = 9.6, 2.3 Hz, 1H).
**$^{19}$F NMR** (376 MHz, Chloroform-*d*) δ -111.36 (m).
Spectroscopic data for **(2c)** is consistent with previously reported data for this compound [8].

**1-Azido-4-chlorobenzene (2d)**

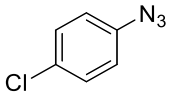


**1-Azido-4-chlorobenzene (2d)** was prepared following method A using 4-chloroaniline (250 mg, 1.9 mmol), sodium nitrite (162 mg, 2.3 mmol), and sodium azide (152 mg, 2.3 mmol). The product was obtained in 70% yield (210 mg) as a yellow solid.

**$^{1}$H NMR** (400 MHz, Chloroform-*d*) δ 7.34–7.29 (m, 2H), 6.98–6.93 (m, 2H).
Spectroscopic data for **(2d)** is consistent with previously reported data for this compound [8].

**1-Azido-4-fluorobenzene (2e)**

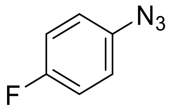


**1-Azido-4-fluorobenzene (2e)** was prepared following method A using 4-fluoroaniline (250 mg, 2.3 mmol), sodium nitrite (186 mg, 2.7 mmol), and sodium azide (176 mg, 2.7 mmol). The product was obtained in 77% yield (237 mg) as a solid.

**$^{1}$H NMR** (400 MHz, Chloroform-*d*) δ 7.09–7.02 (m, 2H), 7.02–6.96 (m, 2H).
**$^{19}$F NMR** (376 MHz, Chloroform-*d*) δ -118.26 (tt, *J* = 8.2, 4.5 Hz).
Spectroscopic data for **(2e)** is consistent with previously reported data for this compound [8].

**5-Azido-1,2,3-trimethoxybenzene (2f)**

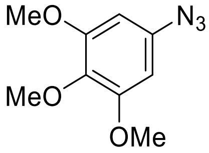


**5-Azido-1,2,3-trimethoxybenzene (2f)** was prepared following method A using 3,4,5-trimethoxyaniline (260 mg, 1.4 mmol), sodium nitrite (117 mg, 1.7 mmol), and sodium azide (111 mg, 1.7 mmol). The product was obtained in 84% yield (249 mg) as a gray solid.

**$^{1}$H NMR** (400 MHz, Chloroform-*d*) δ 6.25 (s, 2H), 3.85 (d, *J* = 0.5 Hz, 6H), 3.81 (d, *J* = 0.6 Hz, 3H).
Spectroscopic data for **(2f)** is consistent with previously reported data for this compound [7].

**4-Azido-1,1'-biphenyl (2g)**

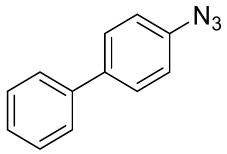


**4-Azido-1,1'-biphenyl (2g)** was prepared following method A using [1,1'-biphenyl]-4-amine (200 mg, 1.2 mmol), sodium nitrite (98 mg, 1.4 mmol), and sodium azide (92 mg, 1.4 mmol). The product was obtained in 87% yield (202 mg) as a brown solid.

**$^1$H NMR** (400 MHz, Chloroform-*d*) δ 7.61–7.54 (m, 4H), 7.44 (ddd, $J$ = 7.8, 6.9, 1.2 Hz, 2H), 7.38–7.32 (m, 1H), 7.13–7.08 (m, 2H).
Spectroscopic data for **(2g)** is consistent with previously reported data for this compound.[9]

**1-(Azidomethyl)-3-(trifluoromethyl)benzene (2h)**

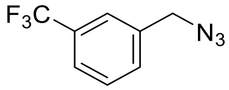


**1-(Azidomethyl)-3-(trifluoromethyl)benzene (2h)** was prepared following method B using 1-(bromomethyl)-3-(trifluoromethyl)benzene (671 mg, 2.8 mmol) and sodium azide (219 mg, 3.4 mmol). The product was obtained in 23% yield (131 mg) as a clear liquid after column chromatography.

**$^1$H NMR** (400 MHz, Chloroform-d) δ 7.6–7.6 (m, 2H), 7.6–7.5 (m, 2H), 4.4 (s, 2H).
**$^{19}$F NMR** (377 MHz, Chloroform-*d*) δ -63.25.
Spectroscopic data for **(2h)** is consistent with previously reported data for this compound [6]**.**

## 6. General procedure for the synthesis of 1,4,5-trisubstituted-1,2,3-triazoles

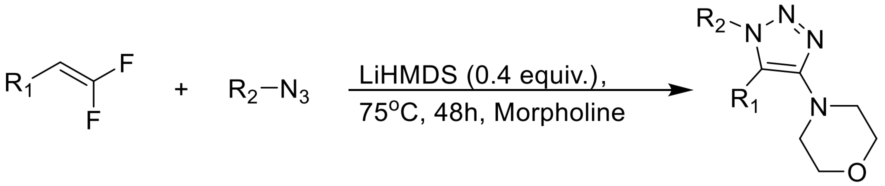


In an oven-dried, Ar-flushed vial with a stirring bar, a solution of the corresponding *gem*-difluoroalkenes (20 mg, 1 equiv) and organic azides (31 mg, 1.5 equiv) were dissolved in morpholine (0.2 mL, 0.4 M) . The solution was stirred for 10 minutes at room temperature under Ar. Then, lithium bis(trimethylsilyl)amide solution (54 µL, 1 M, 0.4 equiv) was added to the reaction mixture and it was purged with Ar three times. The reaction mixture was stirred at 75 °C for 48 h or until completion (monitored by TLC) under the same conditions. Upon completion, brine was added and the organic layer was extracted with ethyl acetate (× 3), dried with anhydrous $Na_2SO_4$, and concentrated *in vacuo*. 4-(5-Morpholino-4-(*p*-tolyl)-1*H*-1,2,3-triazol-1-yl)benzonitrile was obtained after purification by column chromatography (gradient: 0–30% ethyl acetate in hexane) on silica gel.

### 4-(4-Morpholino-5-(*p*-tolyl)-1*H*-1,2,3-triazol-1-yl)benzonitrile (3a)

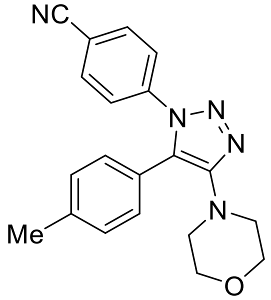

**4-(4-Morpholino-5-(*p*-tolyl)-1*H*-1,2,3-triazol-1-yl)benzonitrile (3a)** was obtained by using 1-(2,2-difluorovinyl)-4-methylbenzene (21 mg, 0.1 mmol), 4-azidobenzonitrile (29 mg, 0.2 mmol) and lithium bis(trimethylsilyl)amide solution (54 µL, 1 M, 0.4 equiv) to obtain product in 70% yield (47 mg) as a light-yellow solid.

**m.p.** 223–225 °C
**$^1$H NMR** (400 MHz, Chloroform-*d*) δ 8.00 (d, *J* = 8.2 Hz, 2H), 7.85 (d, *J* = 8.2 Hz, 2H), 7.50 (d, *J* = 7.6 Hz, 2H), 7.27 (d, *J* = 8.2 Hz, 2H), 3.66 (t, *J* = 4.7 Hz, 4H), 2.93 (t, *J* = 4.6 Hz, 4H), 2.42 (s, 3H).
**$^{13}$C NMR** (126 MHz, Chloroform-*d*) δ 141.5, 139.9, 139.4, 138.5, 133.3, 129.1, 129.0, 127.6, 124.4, 117.7, 112.8, 66.7, 50.6, 21.3.
**HRMS:** $C_{20}H_{19}N_5O$ $[M + H]^+$; calculated 346.1668, found 346.1695.
**IR (ν, cm$^{-1}$):** 2233.05, 2252.44, 2856.26, 2924.22.

**4-(5-(4-(*tert*-Butyl)phenyl)-4-morpholino-1*H*-1,2,3-triazol-1-yl)benzonitrile (3b)**

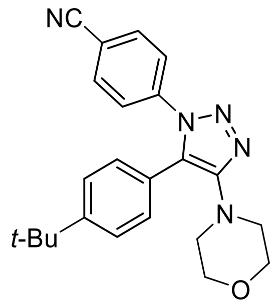


**4-(5-(4-(*tert*-Butyl)phenyl)-4-morpholino-1*H*-1,2,3-triazol-1-yl)benzonitrile (3b)** was obtained by using 1-(tert-butyl)-4-(2,2-difluorovinyl)benzene (23 mg, 0.1 mmol), 4-azidobenzonitrile (25 mg, 0.2 mmol) and lithium bis(trimethylsilyl)amide solution (47 µL, 1 M, 0.4 equiv) to obtain product in 40% yield (18 mg) as a light-yellow solid.

**m.p.** 189–191 $^{o}$C
**$^{1}$H NMR** (400 MHz, Chloroform-*d*) δ 8.01 (d, *J* = 8.9 Hz, 2H), 7.89–7.85 (m, 2H), 7.56 (d, *J* = 8.6 Hz, 2H), 7.49 (d, *J* = 8.7 Hz, 2H), 3.72–3.65 (m, 4H), 2.99–2.91 (m, 4H), 1.37 (s, 9H).
**$^{13}$C NMR** (126 MHz, Chloroform-*d*) δ 151.7, 141.5, 140.0, 133.3, 128.8, 127.6, 125.4, 124.4, 117.8, 112.8, 66.7, 50.7, 34.7, 31.3.
**HRMS:** $C_{23}H_{25}N_5O$ [M + H]$^+$; calculated 388.2137, found 388.2136.
**IR (ν, cm$^{-1}$):** 2212.76, 2229.84, 2856.49, 2975.06, 3065.69, 3102.93.

**4-(5-(4-Methoxyphenyl)-4-morpholino-1*H*-1,2,3-triazol-1-yl)benzonitrile (3c)**

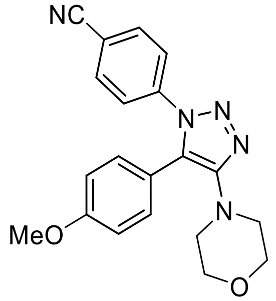


**4-(5-(4-Methoxyphenyl)-4-morpholino-1*H*-1,2,3-triazol-1-yl)benzonitrile (3c)** was obtained by using 1-(2,2-difluorovinyl)-4-methoxybenzene (23 mg, 0.1 mmol), 4-azidobenzonitrile (29 mg, 0.2 mmol) and lithium bis(trimethylsilyl)amide solution (55 µL, 1 M, 0.4 equiv) to obtain product in 51% yield (24 mg) as a light-yellow solid.

**m.p.** 192–194 $^{o}$C
**$^{1}$H NMR** (400 MHz, Chloroform-*d*) δ 8.00 (d, *J* = 8.7 Hz, 2H), 7.86 (d, *J* = 8.7 Hz, 2H), 7.54 (d, *J* = 8.8 Hz, 2H), 7.00 (d, *J* = 8.8 Hz, 2H), 3.87 (s, 3H), 3.69–3.63 (m, 4H), 2.95–2.89 (m, 4H).
**$^{13}$C NMR** (126 MHz, Chloroform-*d*) δ 159.9, 141.3, 139.9, 139.2, 133.3, 130.4, 124.4, 122.9, 117.8, 113.8, 112.7, 66.7, 55.3, 50.7.
**HRMS:** $C_{20}H_{19}N_5O_2$ [M + H]$^+$; calculated 362.1617, found 362.1638.
**IR (ν, cm$^{-1}$):** 2226.45, 2850.60, 2918.34, 3081.67, 3107.08.

**4,4'-(4-Morpholino-1*H*-1,2,3-triazole-1,5-diyl)dibenzonitrile (3d)**

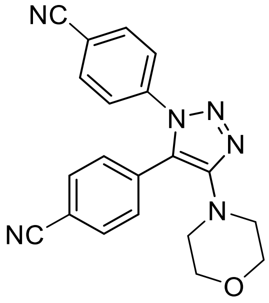


**4,4'-(4-Morpholino-1*H*-1,2,3-triazole-1,5-diyl)dibenzonitrile (3d)** was obtained by using 4-(2,2-difluorovinyl)benzonitrile (22 mg, 0.1 mmol), 4-azidobenzonitrile (29 mg, 0.2 mmol) and lithium bis(trimethylsilyl)amide solution (55 μL, 1 M, 0.4 equiv) to obtain product in 52% yield (25 mg) as a light-yellow solid.

**mp** 258–260 °C
**$^{1}$H NMR** (400 MHz, Chloroform-*d*) δ 7.95–7.87 (m, 4H), 7.84 (d, J = 8.5 Hz, 2H), 7.77 (d, J = 8.4 Hz, 2H), 3.74–3.65 (m, 4H), 2.98–2.90 (m, 4H).
**$^{13}$C NMR** (126 MHz, Chloroform-*d*) δ 142.4, 139.6, 137.0, 135.2, 133.5, 132.3, 128.9, 125.0, 118.5, 117.5, 113.5, 112.1, 66.6, 50.5.
**HRMS:** $C_{20}H_{16}N_5O$ $[M + H]^+$; calculated 358.1492, found 358.1514.
**IR (ν, cm$^{-1}$):** 2226.03, 2826.06, 2849.85, 2859.46, 2893.69, 2915.39, 2961.13, 3063.93, 3082.86, 3100.77.

**4-(4-Morpholino-5-(naphthalen-2-yl)-1*H*-1,2,3-triazol-1-yl)benzonitrile (3e)**

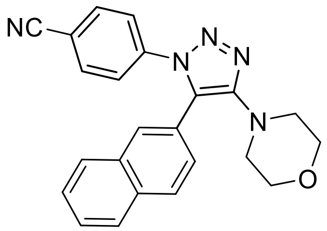


**4-(4-Morpholino-5-(naphthalen-2-yl)-1*H*-1,2,3-triazol-1-yl)benzonitrile (3e)** was obtained by using 2-(2,2-difluorovinyl)naphthalene (22 mg, 0.1 mmol), 4-azidobenzonitrile (25 mg, 0.2 mmol) and lithium bis(trimethylsilyl)amide solution (46 μL, 1 M, 0.4 equiv) to obtain product in 57% yield (25 mg) as a light-yellow solid.

**m.p.** 187–189 °C
**$^{1}$H NMR** (400 MHz, Chloroform-*d*) δ 8.12 (s, 1H), 8.04–7.99 (m, 2H), 7.97–7.87 (m, 5H), 7.76 (dd, J = 8.5, 1.7 Hz, 1H), 7.58–7.53 (m, 2H), 3.72–3.65 (m, 4H), 3.03–2.93 (m, 4H).
**$^{13}$C NMR** (126 MHz, Chloroform-*d*) δ 141.9, 139.9, 139.2, 133.4, 133.1, 133.0, 128.2, 128.1, 127.9, 127.8, 126.7, 126.7, 126.6, 124.6, 117.7, 113.0, 66.7, 50.7, 29.7.
**HRMS:** $C_{23}H_{19}N_5O_2$ $[M + H]^+$; calculated 382.1668, found 382.1694.
**IR (ν, cm$^{-1}$):** 2227.07, 2849.88, 2955.68, 3051.53, 3068.40, 3101.40.

**3-(4-Morpholino-5-(*p*-tolyl)-1*H*-1,2,3-triazol-1-yl)benzonitrile (4a)**

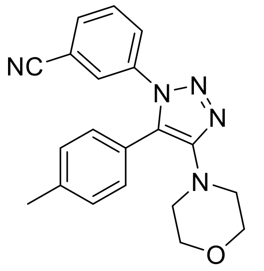


**3-(4-Morpholino-5-(*p*-tolyl)-1*H*-1,2,3-triazol-1-yl)benzonitrile (4a)** was obtained by using 1-(2,2-difluorovinyl)-4-methylbenzene (24 mg, 0.16 mmol), 3-azidobenzonitrile (22 mg, 0.16 mmol) and lithium bis(trimethylsilyl)amide solution (52 µL, 1 M, 0.4 equiv) to obtain product in 41% yield (22 mg) as a white solid.

**m.p.** 169–171 °C
**$^{1}$H NMR** (400 MHz, Chloroform-*d*) δ 8.16 (t, *J* = 1.8 Hz, 1H), 8.07 (ddd, *J* = 8.2, 2.1, 1.2 Hz, 1H), 7.79 (dt, *J* = 7.8, 1.4 Hz, 1H), 7.70 (t, *J* = 8.0 Hz, 1H), 7.54–7.49 (m, 2H), 7.28 (d, *J* = 7.8 Hz, 2H), 3.68–3.65 (m, 4H), 2.96–2.91 (m, 4H), 2.43 (s, 3H).
**$^{13}$C NMR** (126 MHz, Chloroform-*d*) δ 141.4, 139.4, 138.5, 137.3, 132.4, 130.4, 129.2, 129.0, 128.4, 127.7, 127.4, 117.4, 113.7, 66.7, 50.7, 21.3.
**HRMS:** $C_{20}H_{19}N_5O$ $[M + H]^+$; calculated 346.1668, found 346.1695.
**IR (ν, cm$^{-1}$):** 2235.21, 2850.16, 2896.13, 2912.38, 2953.92.

**4-(1-(3,5-Dimethoxyphenyl)-5-(*p*-tolyl)-1*H*-1,2,3-triazol-4-yl)morpholine (4b)**

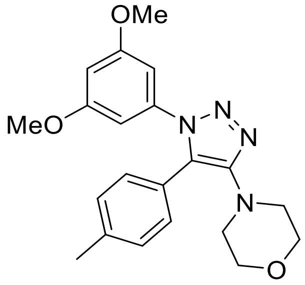


**4-(1-(3,5-Dimethoxyphenyl)-5-(*p*-tolyl)-1*H*-1,2,3-triazol-4-yl)morpholine (4b)** was obtained by using 1-(2,2-difluorovinyl)-4-methylbenzene (22 mg, 0.14 mmol), 1-azido-3,5-dimethoxybenzene (38 mg, 0.21 mmol) and lithium bis(trimethylsilyl)amide solution (52 µL, 1 M, 0.4 equiv) to obtain product in 58% yield (31 mg) as a reddish-brown solid.

**m.p**. 127–129 °C
**$^{1}$H NMR** (400 MHz, Chloroform-*d*) δ 7.64–7.55 (m, 2H), 7.26 (d, *J* = 7.9 Hz, 2H), 6.86 (d, *J* = 2.3 Hz, 2H), 6.57 (t, *J* = 2.3 Hz, 1H), 3.85 (s, 6H), 3.70–3.62 (m, 4H), 2.96–2.90 (m, 4H), 2.41 (s, 3H).
**$^{13}$C NMR** (126 MHz, Chloroform-*d*) δ 161.1, 141.3, 138.0, 129.0, 128.5, 128.1, 126.7, 129.3, 103.2, 101.3, 66.9, 55.7, 50.4, 21.3.
**HRMS:** $C_{21}H_{24}N_4O_3$ $[M + H]^+$; calculated 381.1927, found 381.1941.
**IR (ν, cm$^{-1}$):** 1711.69, 2849.71, 2920.62, 2952.92.

**4-(1-(3-Fluorophenyl)-5-(*p*-tolyl)-1*H*-1,2,3-triazol-4-yl)morpholine (4c)**

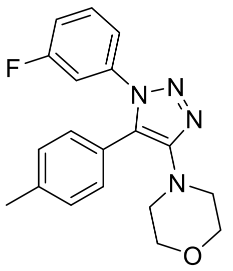

**4-(1-(3-Fluorophenyl)-5-(*p*-tolyl)-1*H*-1,2,3-triazol-4-yl)morpholine (4c)** was obtained by using 1-(2,2-difluorovinyl)-4-methylbenzene (40 mg, 0.26 mmol), 1-azido-3-fluorobenzene (53 mg, 0.39 mmol) and lithium bis(trimethylsilyl)amide solution (104 µL, 1 M, 0.4 equiv) was used to obtain product in 50% yield (44 mg) as a light-yellow solid.

**m.p.** 164–166 $^{o}$C
**$^{1}$H NMR** (400 MHz, Chloroform-*d*) δ 7.59–7.49 (m, 5H), 7.26 (d, *J* = 7.9 Hz, 2H), 7.24–7.18 (m, 1H), 3.70–3.61 (m, 4H), 2.97–2.88 (m, 4H), 2.41 (s, 3H).
**$^{13}$C NMR** (126 MHz, Chloroform-*d*) δ 162.6 (d, *J* = 248.6 Hz), 141.3, 138.7, 138.2, 137.7 (d, *J* = 10.1 Hz), 130.6 (d, *J* = 8.9 Hz), 129.0, 128.7, 127.9, 120.2 (d, *J* = 3.3 Hz), 116.2 (d, *J* = 21.1 Hz), 112.1 (d, *J* = 25.1 Hz), 66.7, 50.5, 21.3.
**$^{19}$F NMR** (376 MHz, Chloroform-*d*) δ -110.80 (td, *J* = 8.8, 5.6 Hz).
**HRMS:** $C_{19}H_{19}FN_4O$ [M + H]$^+$; calculated 339.1621, found 339.1625.
**IR (ν, cm$^{-1}$):** 2849.54, 2894.32, 2916.11, 2950.74, 2965.75.

**4-(1-(4-Chlorophenyl)-5-(*p*-tolyl)-1*H*-1,2,3-triazol-4-yl)morpholine (4d)**

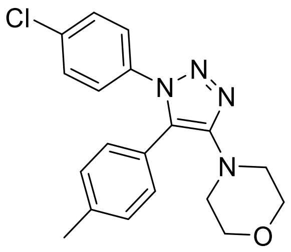

**4-(1-(4-Chlorophenyl)-5-(*p*-tolyl)-1*H*-1,2,3-triazol-4-yl)morpholine (4d)** was obtained by using 1-(2,2-difluorovinyl)-4-methylbenzene (20 mg, 0.14 mmol), 1-azido-4-chlorobenzene and sodium (30 mg, 0.20 mmol) and lithium bis(trimethylsilyl)amide solution (52 µL, 1 M, 0.4 equiv) to obtain product in 39% yield (18 mg) as a light-yellow solid.

**m.p.** 205–207 $^{o}$C
**$^{1}$H NMR** (400 MHz, Methanol-*d*$_4$) δ 7.82–7.76 (m, 2H), 7.72–7.66 (m, 2H), 7.57 (d, *J* = 8.0 Hz, 2H), 7.36 (d, *J* = 7.7 Hz, 2H), 3.68–3.59 (m, 4H), 2.98–2.89 (m, 4H), 2.45 (s, 3H).
**$^{13}$C NMR** (126 MHz, Chloroform-*d*) δ 141.4, 138.7, 138.2, 135.2, 135.1, 129.5, 129.1, 128.7, 128.0, 125.9, 66.8, 50.5, 21.3.
**HRMS:** $C_{19}H_{19}ClN_4O$ [M + H]$^+$; calculated 355.1326, found 355.1330.
**IR (ν, cm$^{-1}$):** 2851.68, 2886.79, 2941.53, 2963.04.

**4-(1-(4-Fluorophenyl)-5-(*p*-tolyl)-1*H*-1,2,3-triazol-4-yl)morpholine (4e)**

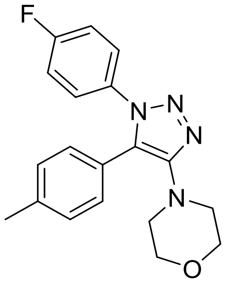


**4-(1-(4-Fluorophenyl)-5-(*p*-tolyl)-1*H*-1,2,3-triazol-4-yl)morpholine (4e)** was obtained by using 1-(2,2-difluorovinyl)-4-methylbenzene (40 mg, 0.26 mmol), 1-azido-4-fluorobenzene (53 mg, 0.39 mmol), and lithium bis(trimethylsilyl)amide solution (105 µL, 1 M, 0.4 equiv) to obtain product in 22% yield (19 mg) as a light-yellow solid.

**4-(1-(4-Fluorophenyl)-5-(*p*-tolyl)-1*H*-1,2,3-triazol-4-yl)morpholine (4e)** was obtained by using 1-(2,2-difluorovinyl)-4-methylbenzene (23 mg, 0.14 mmol), 1-azido-4-fluorobenzene (30 mg, 0.22 mmol), copper(II) sulfate anhydrous (23 mg, 0.14 mmol) and lithium bis(trimethylsilyl)amide solution (105 µL, 1 M, 0.4 equiv) to obtain product in 56% yield (28 mg) as a light-yellow solid.

**m.p.** 177–179 °C
**$^1$H NMR** (400 MHz, Chloroform-*d*) δ 7.72–7.65 (m, 2H), 7.62–7.56 (m, 2H), 7.30–7.20 (m, 4H), 3.68–3.59 (m, 4H), 2.94–2.86 (m, 4H), 2.41 (s, 3H).
**$^{19}$F NMR** (376 MHz, Chloroform-*d*) δ -111.55 (ddd, *J* = 12.9, 8.2, 4.7 Hz).
**$^{13}$C NMR** (126 MHz, Chloroform-*d*) δ 162.7 (d, *J* = 250.2 Hz), 141.4, 138.5, 138.1, 132.7 (d, *J* = 3.2 Hz), 129.1, 128.5, 128.0, 126.8 (d, *J* = 8.7 Hz), 116.3 (d, *J* = 23.2 Hz), 66.8, 50.5, 21.3.
**HRMS:** $C_{19}H_{19}FN_4O$ [M + H]$^+$; calculated 339.1621, found 339.1651.
**IR (ν, cm$^{-1}$):** 2332.47, 2360.85, 2848.90, 2890.26, 2912.21, 2946.79, 3075.27.

**4-(5-(*p*-Tolyl)-1-(3,4,5-trimethoxyphenyl)-1*H*-1,2,3-triazol-4-yl)morpholine (4f)**

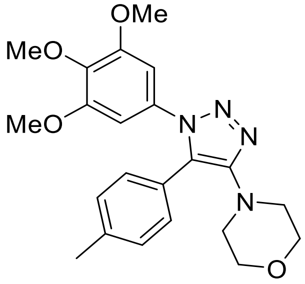


**4-(5-(*p*-Tolyl)-1-(3,4,5-trimethoxyphenyl)-1*H*-1,2,3-triazol-4-yl)morpholine (4f)** was obtained by using 1-(2,2-difluorovinyl)-4-methylbenzene (22 mg, 0.14 mmol), 5-azido-1,2,3-trimethoxybenzene (29 mg, 0.14 mmol) and lithium bis(trimethylsilyl)amide solution (52µL, 1 M, 0.4 equiv) to obtain product in 36% yield (21 mg) as yellowish-brown solid.

**m.p.** 150–152 °C

**$^1$H NMR** (400 MHz, Chloroform-*d*) δ 7.63–7.57 (m, 2H), 7.31–7.22 (m, 2H), 6.93 (s, 2H), 3.92 (s, 9H), 3.70–3.61 (m, 4H), 2.97–2.90 (m, 4H), 2.41 (s, 3H).

**$^{13}$C NMR** (126 MHz, Chloroform-*d*) δ 153.5, 141.3, 138.6, 138.1, 138.0, 132.2, 129.1, 128.5, 128.2, 102.6, 67.0, 61.1, 56.4, 50.4, 21.3.

**HRMS:** $C_{21}H_{24}N_4O_3$ $[M + H]^+$; calculated 411.2032, found 411.2045.

**IR (ν, cm$^{-1}$):** 2847.85, 2918.43, 2949.23, 3002.17.

**4-(1-([1,1'-Biphenyl]-4-yl)-5-(*p*-tolyl)-1*H*-1,2,3-triazol-4-yl)morpholine (4g)**

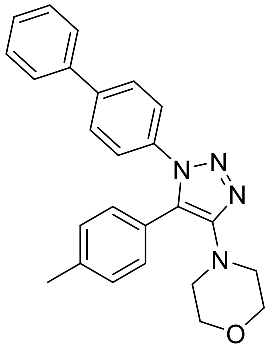


**4-(1-([1,1'-Biphenyl]-4-yl)-5-(*p*-tolyl)-1*H*-1,2,3-triazol-4-yl)morpholine (4g)** was obtained by using 1-(2,2-difluorovinyl)-4-methylbenzene (20 mg, 0.13 mmol), 4-azido-1,1'-biphenyl (38 mg, 0.19 mmol) and lithium bis(trimethylsilyl)amide solution (52 µL, 1 M, 0.4 equiv) to obtain product in 31% yield (12 mg) as a light-yellow solid.

**m.p.** 202-204 °C

**$^1$H NMR** (400 MHz, Chloroform-*d*) δ 7.78 (s, 4H), 7.68–7.61 (m, 4H), 7.53–7.47 (m, 2H), 7.43 (dd, *J* = 6.9, 1.8 Hz, 1H), 7.28 (d, *J* = 7.9 Hz, 2H), 3.70–3.65 (m, 4H), 2.99–2.94 (m, 4H), 2.42 (s, 3H).

**$^{13}$C NMR** (126 MHz, Chloroform-*d*) δ 142.2, 141.4, 139.7, 138.5, 138.0, 135.7, 129.1, 129.0, 128.5, 128.2, 128.0, 127.9, 127.2, 125.1, 66.9, 50.5, 21.3.

**HRMS:** $C_{25}H_{24}N_4O$ $[M + H]^+$; calculated 397.2029, found 397.2039

**IR (ν, cm$^{-1}$):** 2349.28, 2846.10, 2920.73, 3288.72

**4-(5-(*p*-Tolyl)-1-(3-(trifluoromethyl)benzyl)-1*H*-1,2,3-triazol-4-yl)morpholine (4h)**

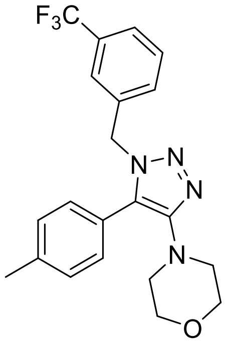

**4-(5-(*p*-Tolyl)-1-(3-(trifluoromethyl)benzyl)-1*H*-1,2,3-triazol-4-yl)morpholine (4h)** was obtained by using 1-(2,2-difluorovinyl)-4-methylbenzene (21 mg, 0.14 mmol), 1-(azidomethyl)-3-(trifluoromethyl)benzene (42 mg, 0.21 mmol) and lithium bis(trimethylsilyl)amide solution (56 µL, 1 M, 0.4 equiv) at 110 $^{o}$C for 72h to obtain product in 44% yield (25 mg) as a clear sticky liquid.

**$^{1}$H NMR** (400 MHz, Chloroform-*d*) δ 7.63–7.57 (m, 2H), 7.55–7.47 (m, 4H), 7.24 (d, *J* = 7.9 Hz, 2H), 5.53 (s, 2H), 3.71–3.63 (m, 4H), 2.86–2.79 (m, 4H), 2.39 (s, 3H).
**$^{19}$F NMR** (376 MHz, Chloroform-*d*) δ -63.24.
**$^{13}$C NMR** (126 MHz, Chloroform-*d*) δ 140.9, 140.7, 138.2, 136.7, 131.3 (q, *J* = 32.8 Hz), 130.9, 129.6, 129.1, 128.6, 128.0, 125.2 (q, *J* = 3.7 Hz), 124.4 (q, *J* = 3.7 Hz), 123.7 (q, *J* = 272.3 Hz), 67.0, 50.9, 50.3, 21.3.
**HRMS:** $C_{21}H_{21}F_3N_4O$ [M + H]$^+$; calculated 403.1746, found 403.1750.
**IR (ν, cm$^{-1}$):** 1720.11, 2855.35, 2920.86, 2958.44.

**4-(4-(Piperidin-1-yl)-5-(*p*-tolyl)-1*H*-1,2,3-triazol-1-yl)benzonitrile (5a)**

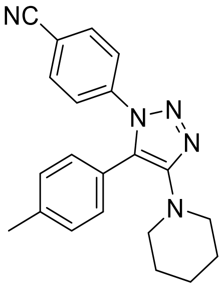

**4-(4-(Piperidin-1-yl)-5-(*p*-tolyl)-1*H*-1,2,3-triazol-1-yl)benzonitrile (5a)** was obtained by using 1-(2,2-difluorovinyl)-4-methylbenzene 1-(2,2-difluorovinyl)-4-methylbenzene (22.33 mg, 1 equiv, 144.8 µmol) and 4-azidobenzonitrile (31.32 mg, 1.5 equiv, 217.3 µmol) dissolved in piperidine (0.32 mL, 0.38 M). The reaction mixture ran with Ar and stirred for 10 minutes at room temperature. Then, lithium bis(trimethylsilyl)amide solution (57.94 µL, 1 molar, 0.4 equiv) was added to the mixture and it was purged with Ar (× 3). Then, the mixture was heated to

75 °C and stirred for 48 h to obtain the product with 42% yield (21 mg) as white solid after purification by column chromatography.

**m.p.** 175–177 °C
**$^{1}$H NMR** (400 MHz, Chloroform-*d*) δ 8.01 (d, *J* = 8.8 Hz, 2H), 7.84 (d, *J* = 8.8 Hz, 2H), 7.50 (d, *J* = 8.1 Hz, 2H), 7.29 – 7.27 (m, 1H), 7.25 (d, *J* = 1.1 Hz, 1H), 2.86 (s, 4H), 2.42 (s, 3H), 1.53 (s, 6H).
**$^{13}$C NMR** (126 MHz, Chloroform-d) δ 142.9, 140.3, 139.0, 138.1, 133.2, 129.0, 128.9, 128.2, 124.2, 118.0, 112.4, 51.9, 25.8, 23.5, 21.3.
**HRMS:** $C_{21}H_{21}N_5$ $[M + H]^+$; calculated 344.1875, found 344.1858
**IR (ν, cm$^{-1}$):** 1693.13, 2229.17, 2853.22, 2924.54

**4-(4-(Azepan-1-yl)-5-(*p*-tolyl)-1*H*-1,2,3-triazol-4-yl)benzonitrile (5b)**

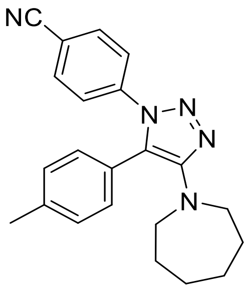


**4-(4-(Azepan-1-yl)-5-(*p*-tolyl)-1*H*-1,2,3-triazol-4-yl)benzonitrile (5b)** was obtained by using 1-(2,2-difluorovinyl)-4-methylbenzene (20.54 mg, 1 equiv, 133.2 µmol) and 4-azidobenzonitrile (28.81 mg, 1.5 equiv, 199.9 µmol) dissolved in hexamethyleneimine (0.300 mL, 0.4 M). The reaction mixture was run with Ar and stirred for 10 minutes at room temperature. Then, lithium bis(trimethylsilyl)amide solution (53.30 µL, 1 molar, 0.4 equiv) was added to the mixture and it was purged with Ar (× 3). Then, the mixture was heated to 75 °C and stirred for 48 h to obtain the product with 30% yield (14.2 mg) as white solid after purification by column chromatography.

**m.p.** 158–160 °C
**$^{1}$H NMR** (400 MHz, Chloroform-*d*) δ 7.92 – 7.82 (m, 4H), 7.59 – 7.55 (m, 2H), 7.27 (d, *J* = 7.6 Hz, 2H), 3.13 – 3.00 (m, 4H), 2.41 (s, 3H), 1.6 (s, 8H).
**$^{13}$C NMR** (126 MHz, Chloroform-*d*) δ 143.9, 140.1, 138.9, 138.1, 133.2, 129.2, 128.0, 127.9, 125.1, 118.0, 112.7, 54.1, 29.5, 27.7, 21.4.
**HRMS:** $C_{22}H_{23}N_5$ $[M + H]^+$; calculated 358.2032, found 358.2007
**IR (ν, cm$^{-1}$):** 2218.62, 2877.88, 2950.55, 3030.90.

Table S2: Difference between the calculated and the observed HRMS data.

| No. | MF$[M + H]^+$ | Calculated | Found | Difference |
|---|---|---|---|---|
| 3a | $C_{20}H_{19}N_5O$ | 346.1668 | 346.1695 | 0.0027 |
| 3b | $C_{23}H_{25}N_5O$ | 388.2137 | 388.2136 | 0.0001 |
| 3c | $C_{20}H_{19}N_5O_2$ | 362.1617 | 362.1638 | 0.0021 |
| 3d | $C_{20}H_{16}N_5O$ | 358.1492 | 358.1514 | 0.0022 |
| 3e | $C_{23}H_{19}N_5O_2$ | 382.1668 | 382.1694 | 0.0026 |
| 4a | $C_{20}H_{19}N_5O$ | 346.1668 | 346.1695 | 0.0027 |
| 4b | $C_{21}H_{24}N_4O_3$ | 381.1927 | 381.1941 | 0.0014 |
| 4c | $C_{19}H_{19}FN_4O$ | 339.1621 | 339.1625 | 0.0004 |
| 4d | $C_{19}H_{19}ClN_4O$ | 355.1326 | 355.1330 | 0.0004 |
| 4e | $C_{19}H_{19}FN_4O$ | 339.1621 | 339.1651 | 0.0030 |
| 4f | $C_{21}H_{24}N_4O_3$ | 411.2032 | 411.2045 | 0.0013 |
| 4g | $C_{25}H_{24}N_4O$ | 397.2029 | 397.2039 | 0.0010 |
| 4h | $C_{21}H_{21}F_3N_4O$ | 403.1746 | 403.1750 | 0.0004 |
| 5a | $C_{21}H_{21}N_5$ | 344.1875 | 344.1858 | 0.0017 |
| 5b | $C_{22}H_{23}N_5$ | 358.2032 | 358.2007 | 0.0025 |

## 7. Time course study

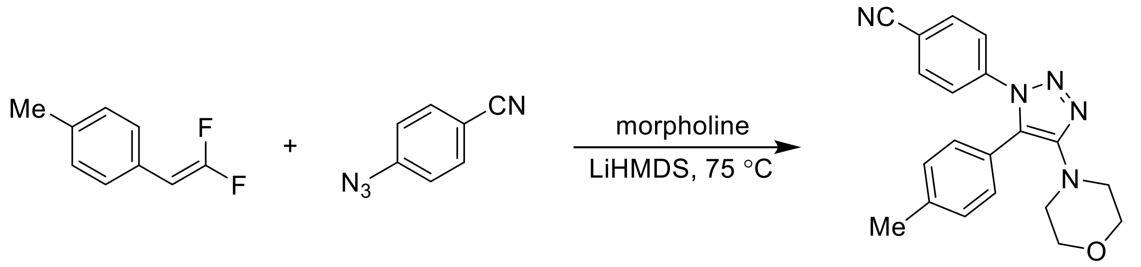


**1** **2b** **3a**

In an oven-dried, Ar-flushed vial with a stirring bar, a solution of 1-(2,2-difluorovinyl)-4-methylbenzene (**1**, 30 mg, 1 equiv) and 4-azidobenzonitrile (**2b**, 42 mg, 1.5 equiv) were dissolved in morpholine (0.3 mL, 0.4 M). The solution was stirred for 10 minutes at room temperature under Ar. Then, LiHMDS (78 µL, 1 M in THF, 0.4 equiv) was added to the reaction mixture and it was purged with Ar three times. The reaction mixture was subjected to continuous stirring at a temperature of 75 °C. After 30 min, 1 h, 2 h, 4 h, 8 h, 16 h, 24 h, 32 h, and 48 h, 50 µL aliquots of the reaction mixture were withdrawn using a syringe. The progress of the reaction was monitored by $^{19}$F NMR (Figure 3 in main text).

## 8. Mechanistic study

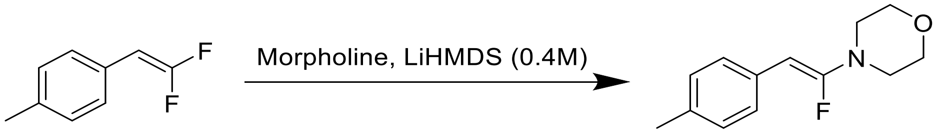


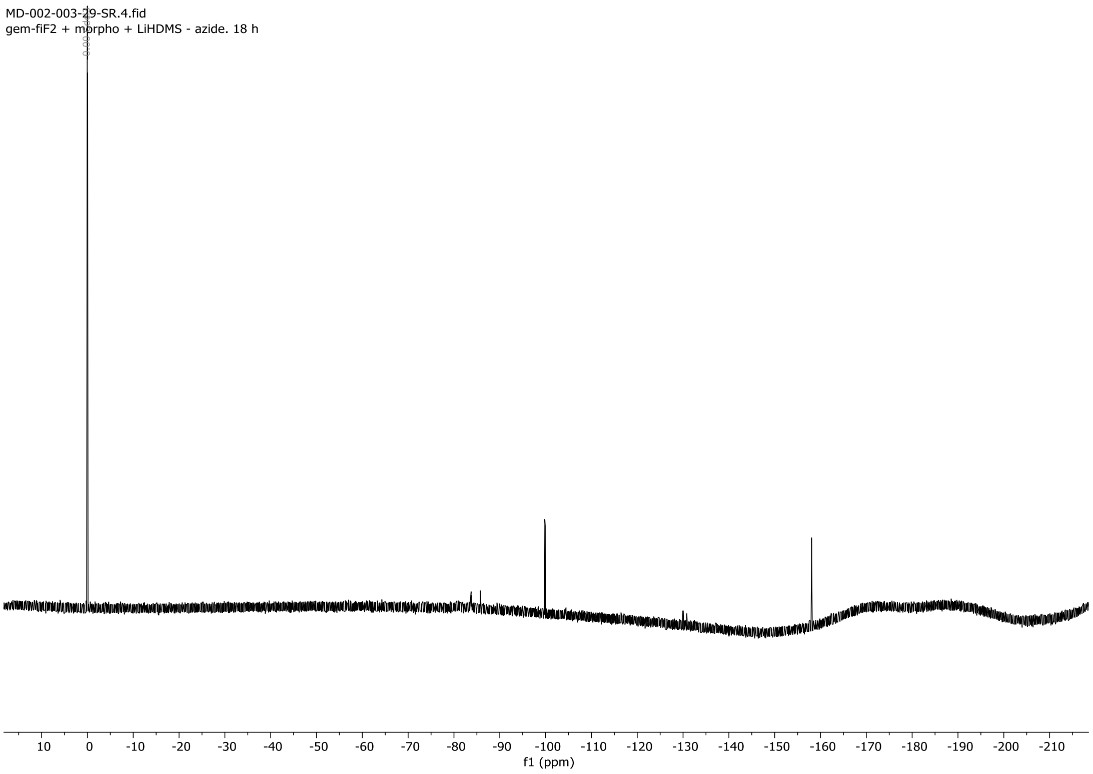


**Figure S3**. Aliquot $^{19}$F NMR.

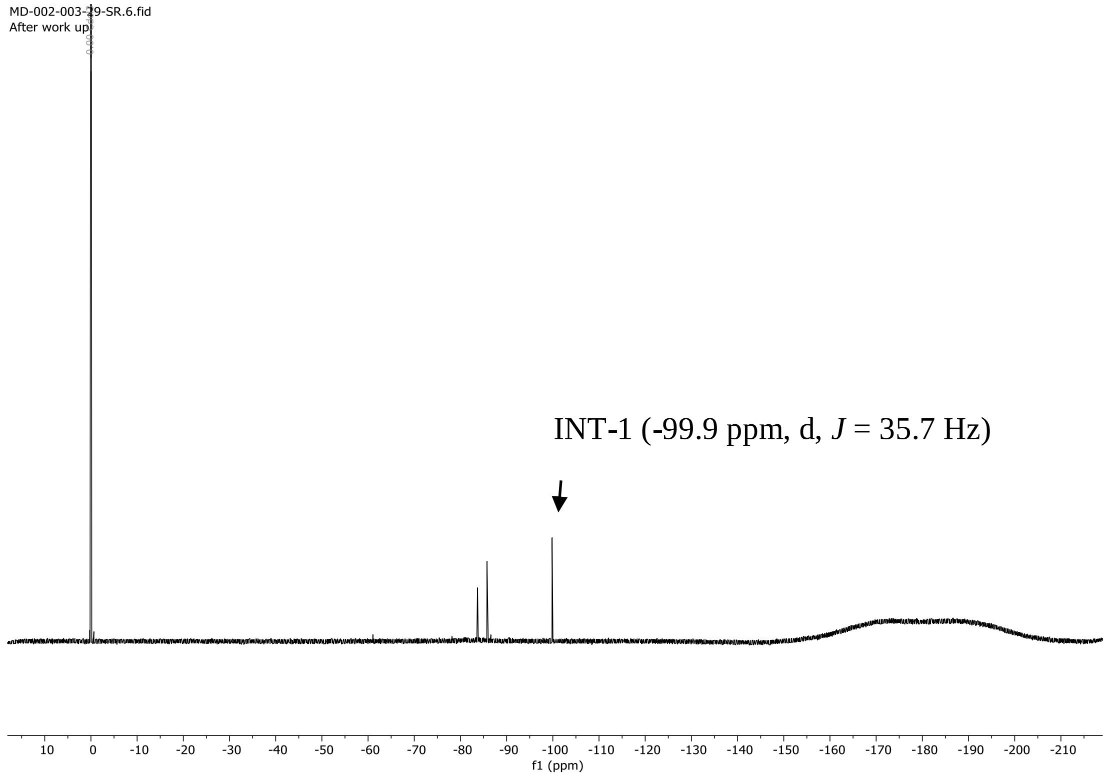


**Figure S4**. Crude $^{19}$F NMR after work-up.

We also performed an energy minimization study using Chem3D to elucidate the conformation of the intermediate, **INT-1**. We found the *Z*-conformation (17.1988 kcal/mol) to be more stable than the *E*-geometry (24.9361 kcal/mol).

*Z*-conformation: 17.1988 kcal/mol

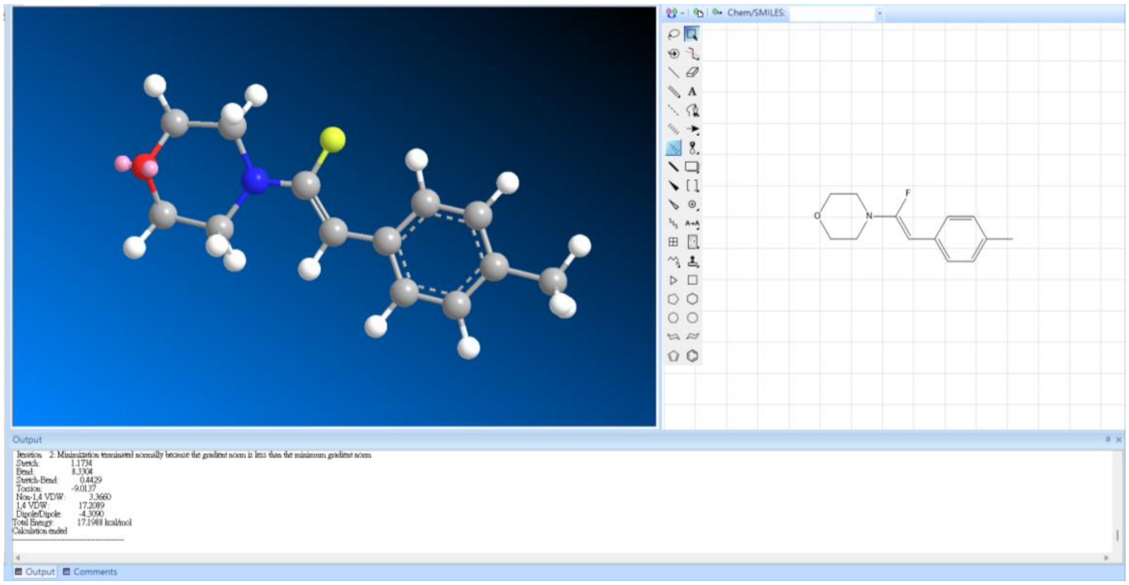


**Figure S5**. Energy minimization study of *Z*-conformation.

*E*-conformation: 24.9361 kcal/mol

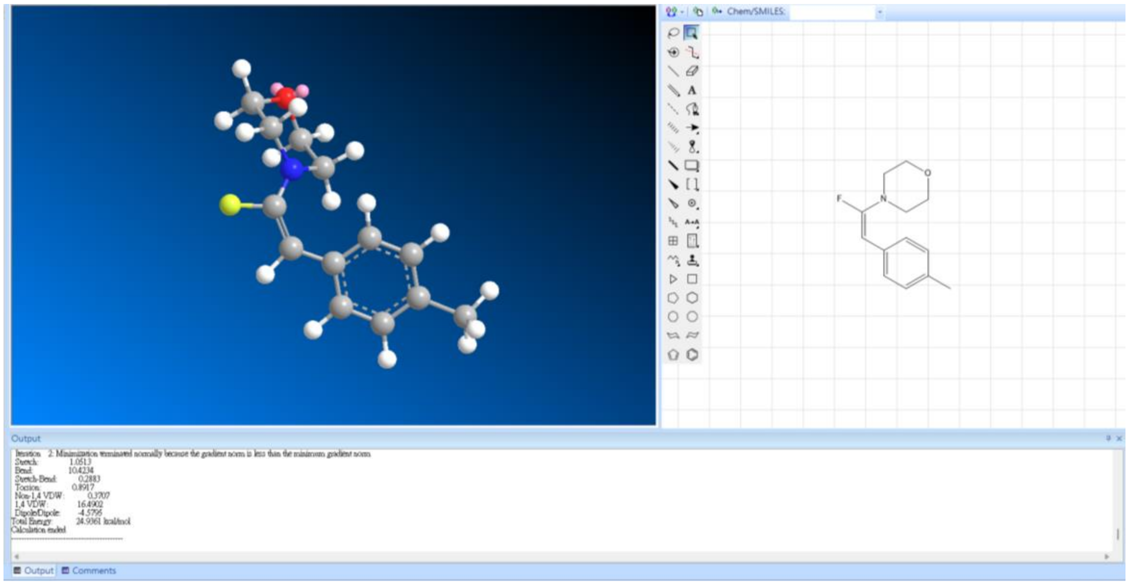


**Figure S6**. Energy minimization study of *E*-conformation.

## 9. Regioisomer study:

The 3D structure of the 1,5-regioisomer (energy-minimized) reflecting the distance between the $H_1$ proton (labeled as yellow) and the protons in morpholine ($H_a$, $H_a'$ and $H_b$, $H_b'$) protons in morpholine:

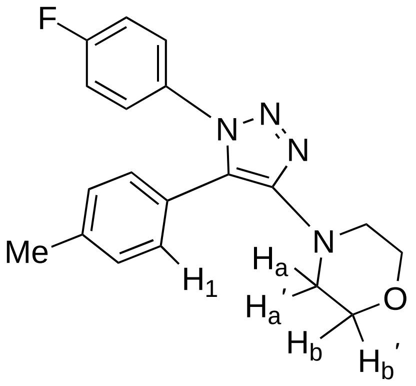


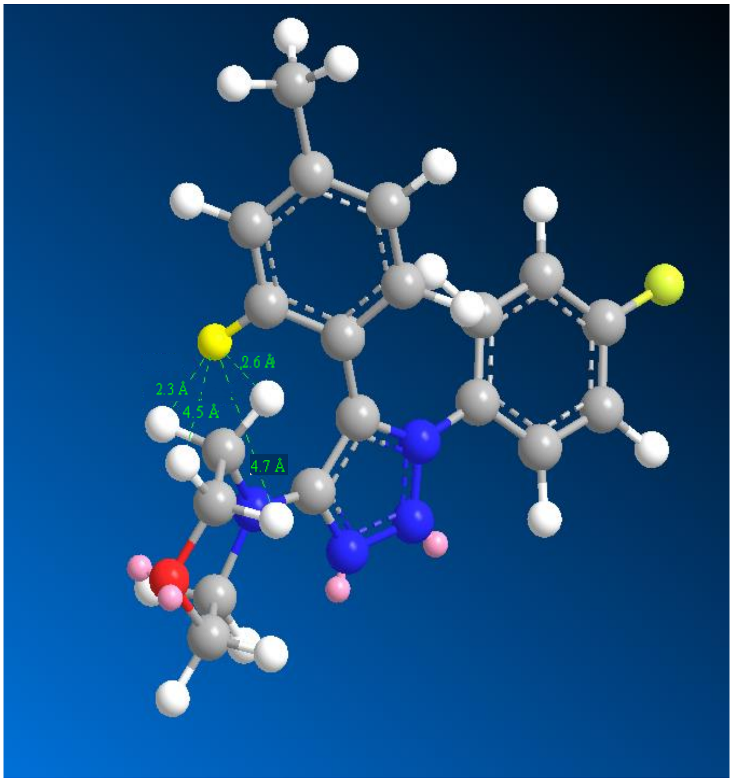


**Figure S7**. 3D structure of 1,5-regioisomer

The 3D structure of the 1,4-regioisomer (energy-minimized) reflecting the distance between the $H_1$ proton (labeled as yellow) and the protons in morpholine ($H_a$, $H_a'$ and $H_b$, $H_b'$) protons in morpholine:

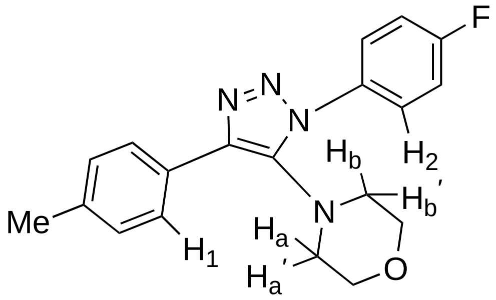


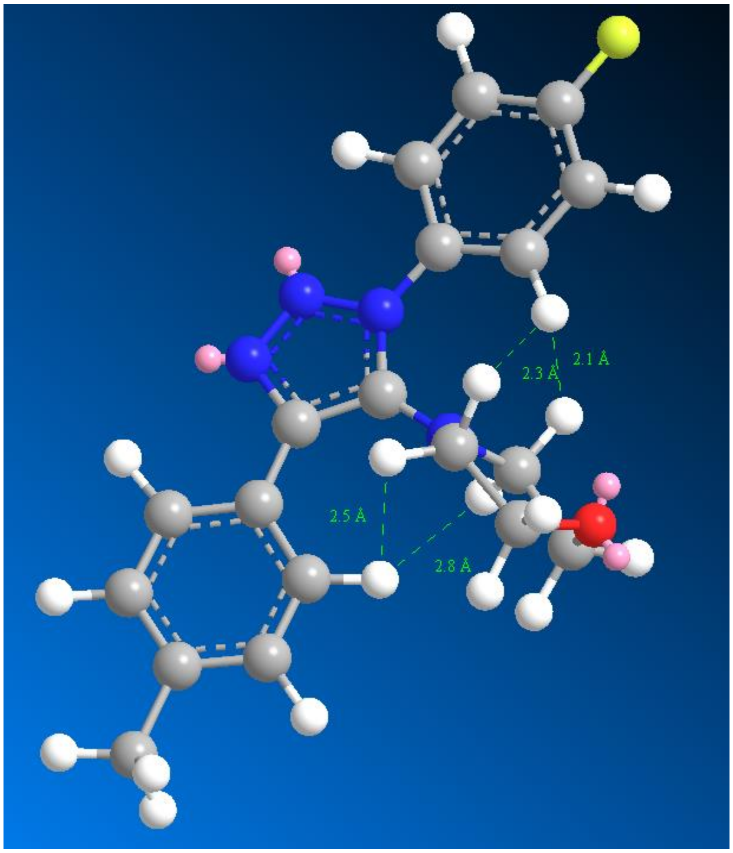


**Figure S8**. 3D structure of 1,4-regioisomer

## 10. Scale-up experiment:

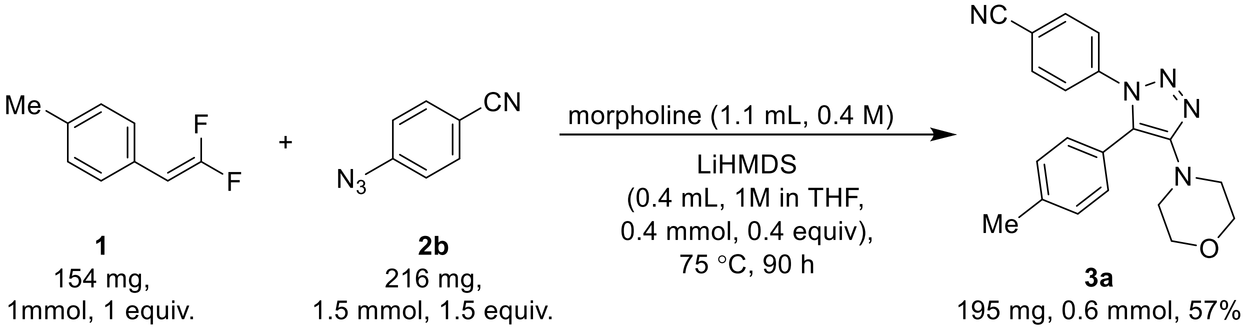


**1**
154 mg,
1mmol, 1 equiv.

**2b**
216 mg,
1.5 mmol, 1.5 equiv.

**3a**
195 mg, 0.6 mmol, 57%

In an oven-dried, Ar-flushed round-bottomed flask with a stirring bar, a solution of 1-(2,2-difluorovinyl)-4-methylbenzene (**1**, 154 mg, 1 equiv) and 4-azidobenzonitrile (**2b**, 108 mg, 0.75 equiv) were dissolved in morpholine (1.1 mL, 0.4 M). The solution was stirred for 10 minutes at room temperature under Ar. Then, lithium bis(trimethylsilyl)amide solution (0.4 mL, 1 M in THF, 0.4 equiv) was added to the reaction mixture and it was purged with Ar three times. The reaction mixture was stirred at 75 °C. After 16 h, another portion of 4-azidobenzonitrile (**2b**, 108 mg, 0.75 equiv) was dissolved in morpholine (1.1 mL, 0.4 molar) and added to the reaction mixture. The reaction was monitored by TLC under the same conditions. Upon completion after 90 h, brine was added and the organic layer was extracted with ethyl acetate (× 3), dried with anhydrous $Na_2SO_4$, and concentrated in vacuo. 4-(5-Morpholino-4-(*p*-tolyl)-1*H*-1,2,3-triazol-1-yl)benzonitrile was obtained in 57% yield after purification by column chromatography (gradient: 0–30% ethyl acetate in hexane).

## 11. $^{1}$H, $^{19}$F and $^{13}$C NMR spectra:

### $^{1}$H NMR of 3a

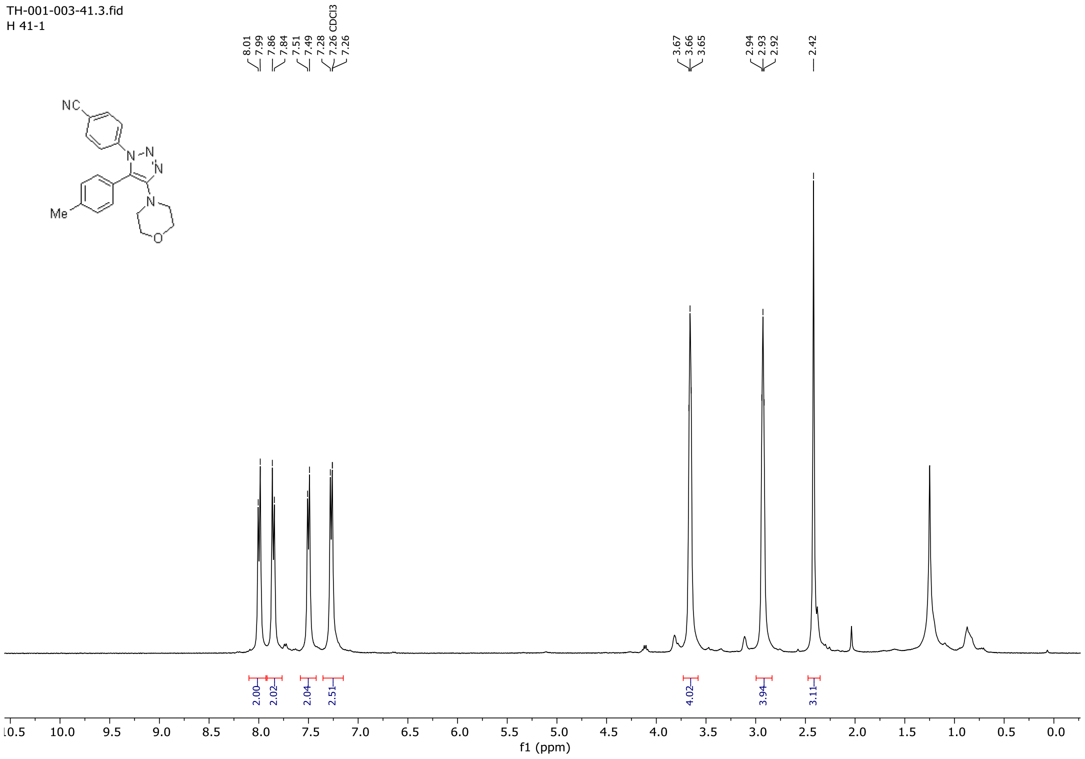


### $^{13}$C NMR of 3a

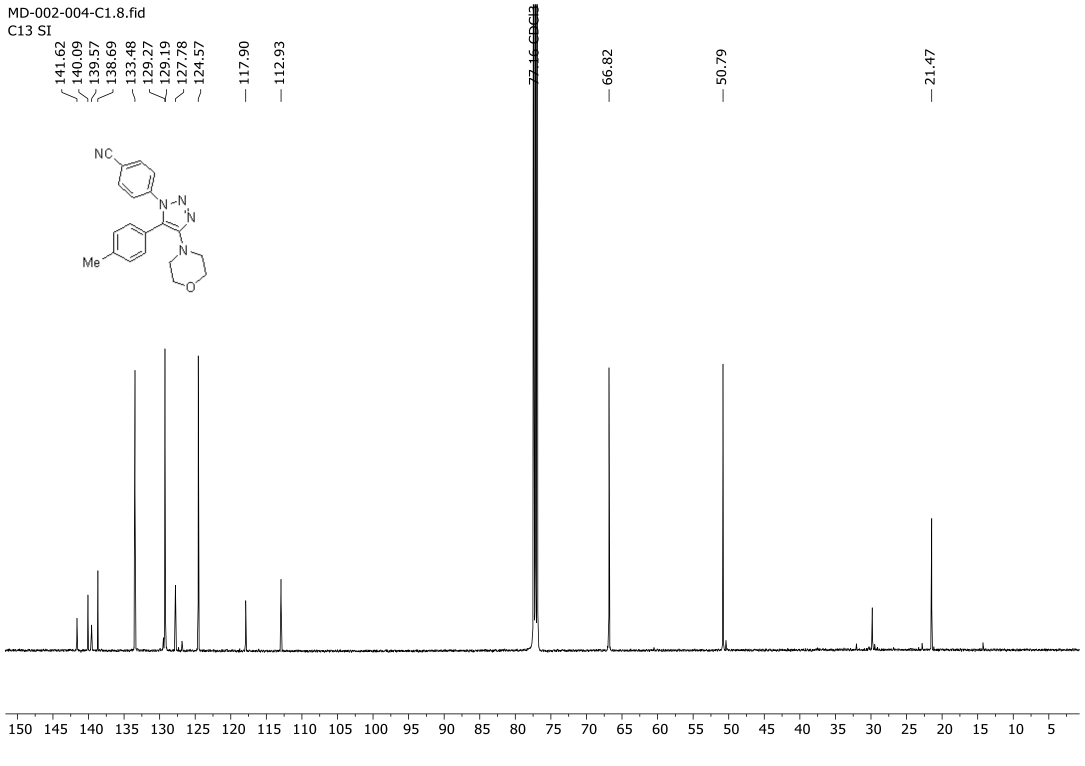

# $^{1}$H NMR of 3b

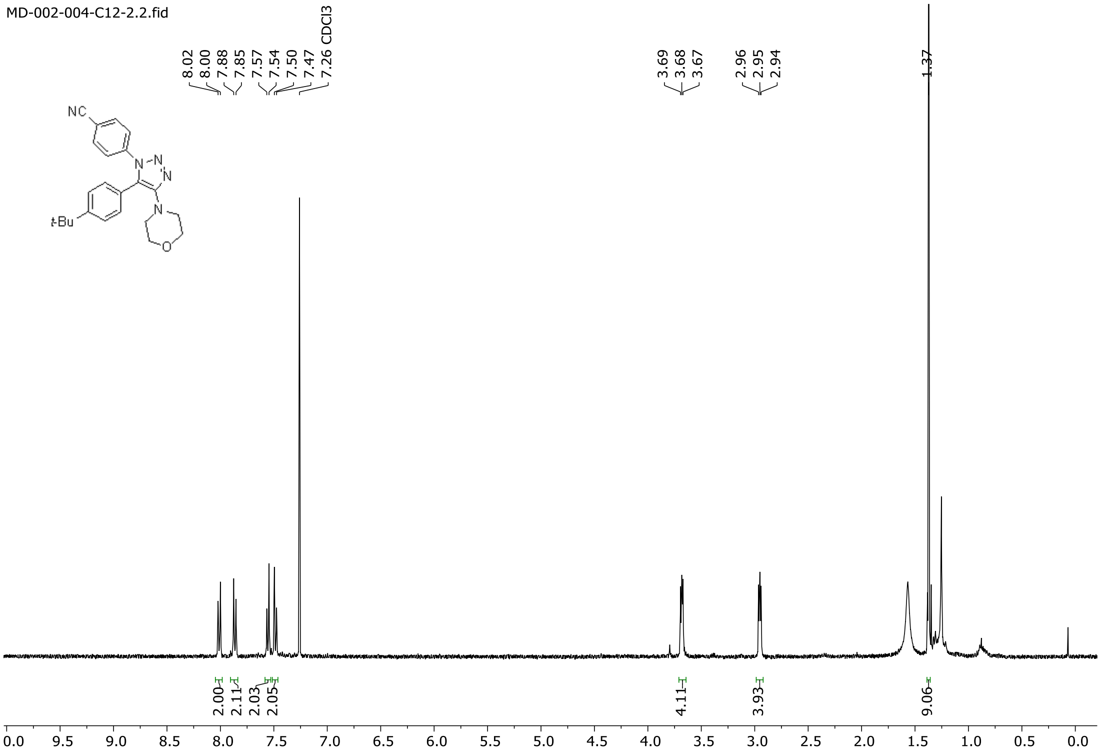


# $^{13}$C NMR of 3b

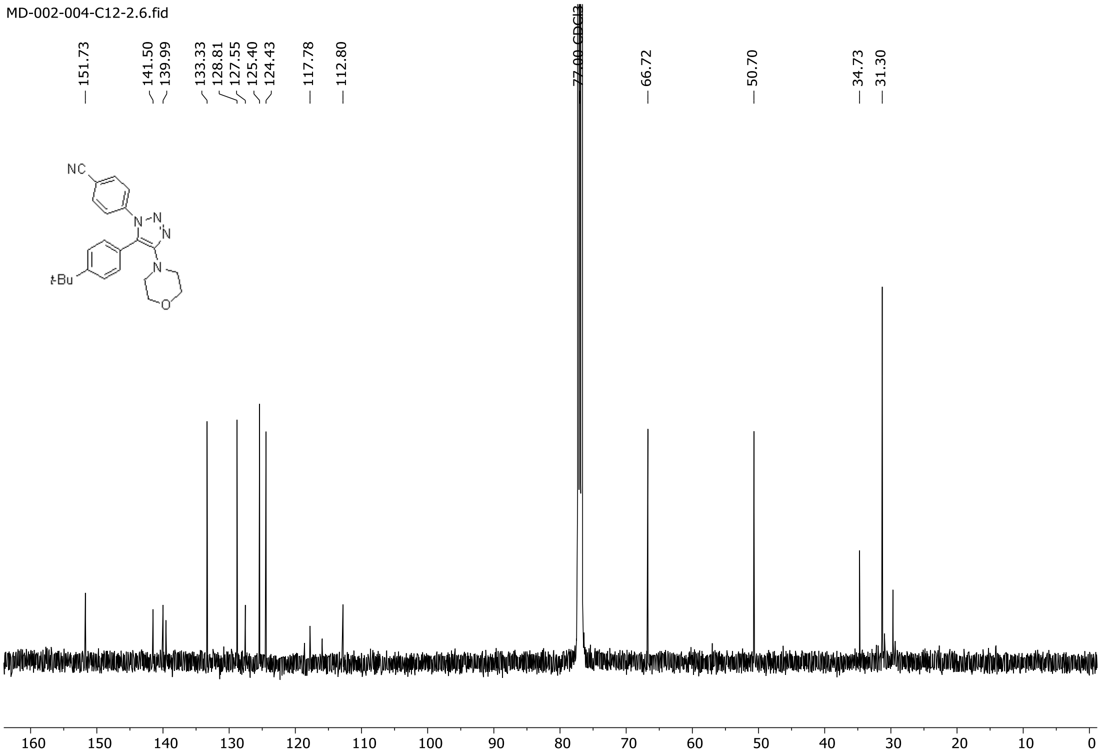

## $^{1}$H NMR of 3c

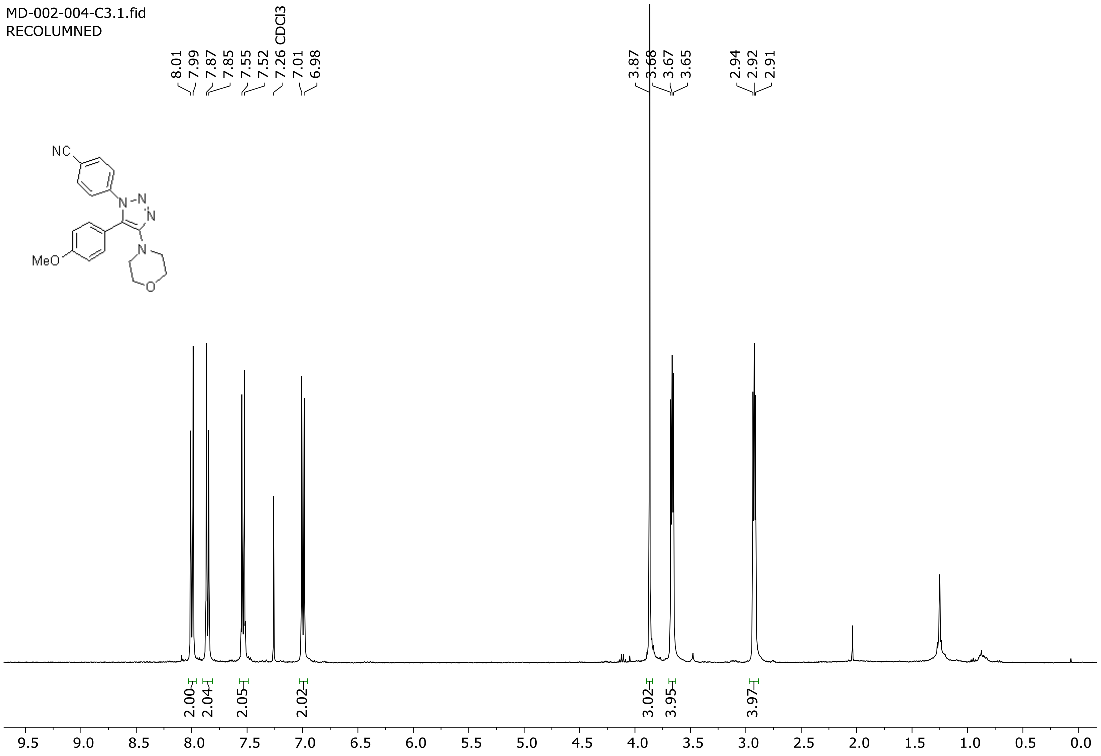


## $^{13}$C NMR of 3c

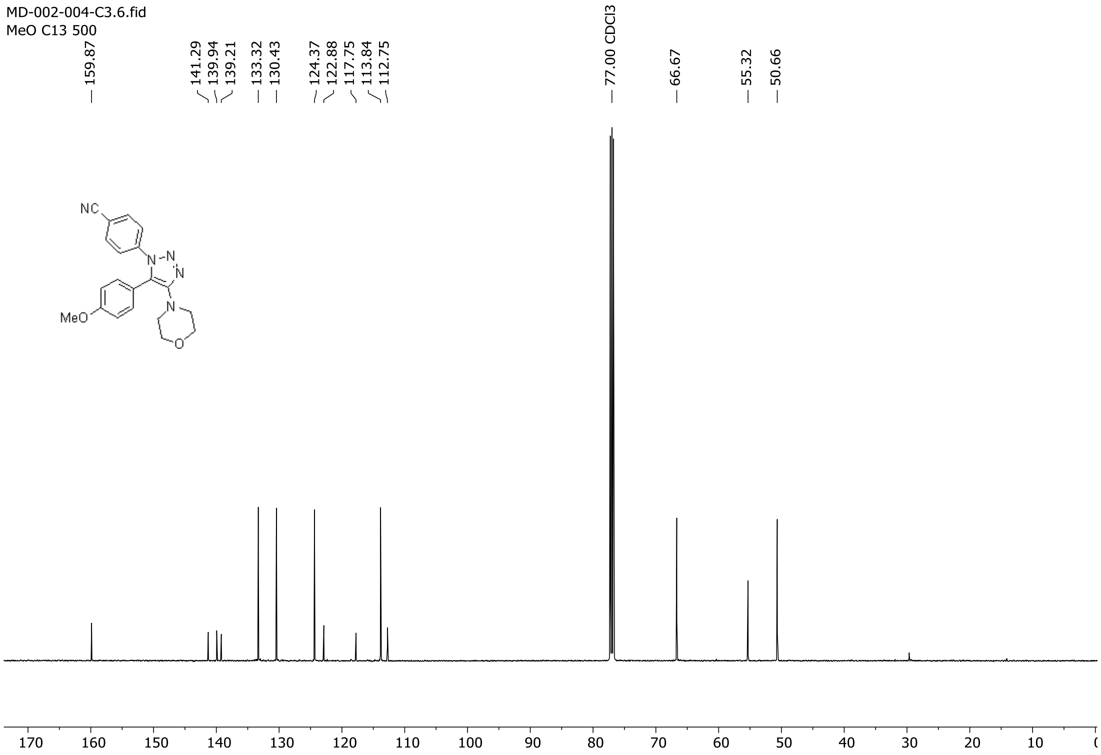

# $^{1}$H NMR of 3d

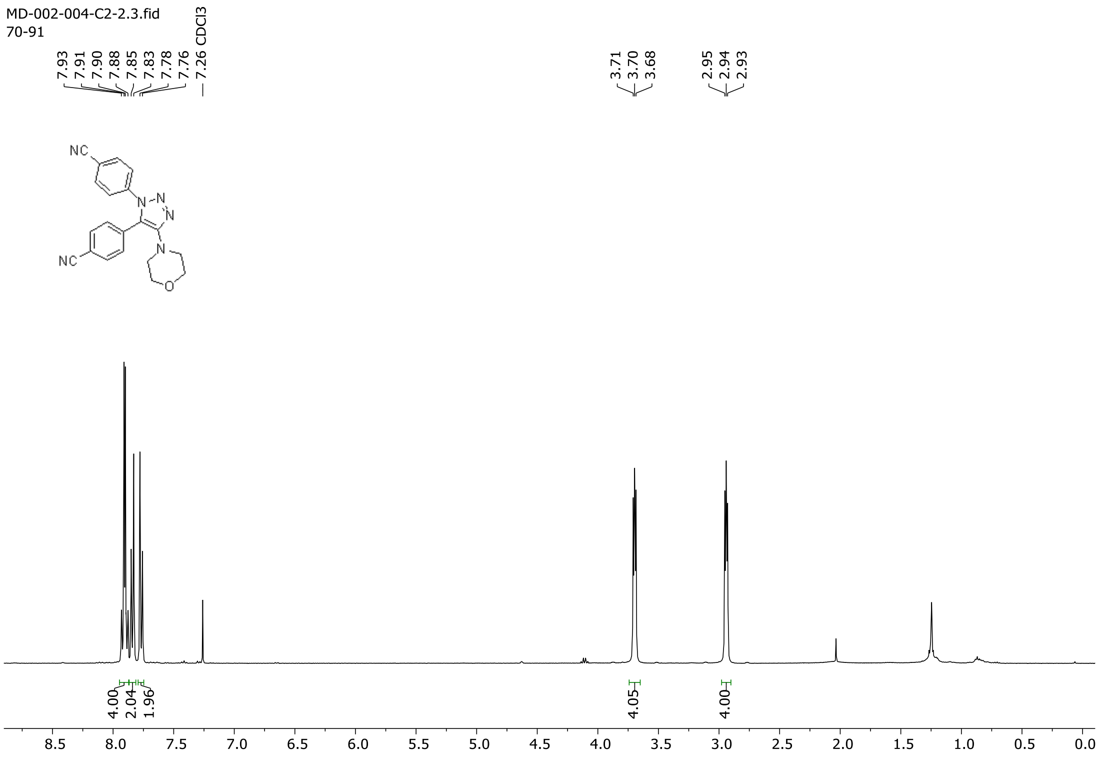


# $^{13}$C NMR of 3d

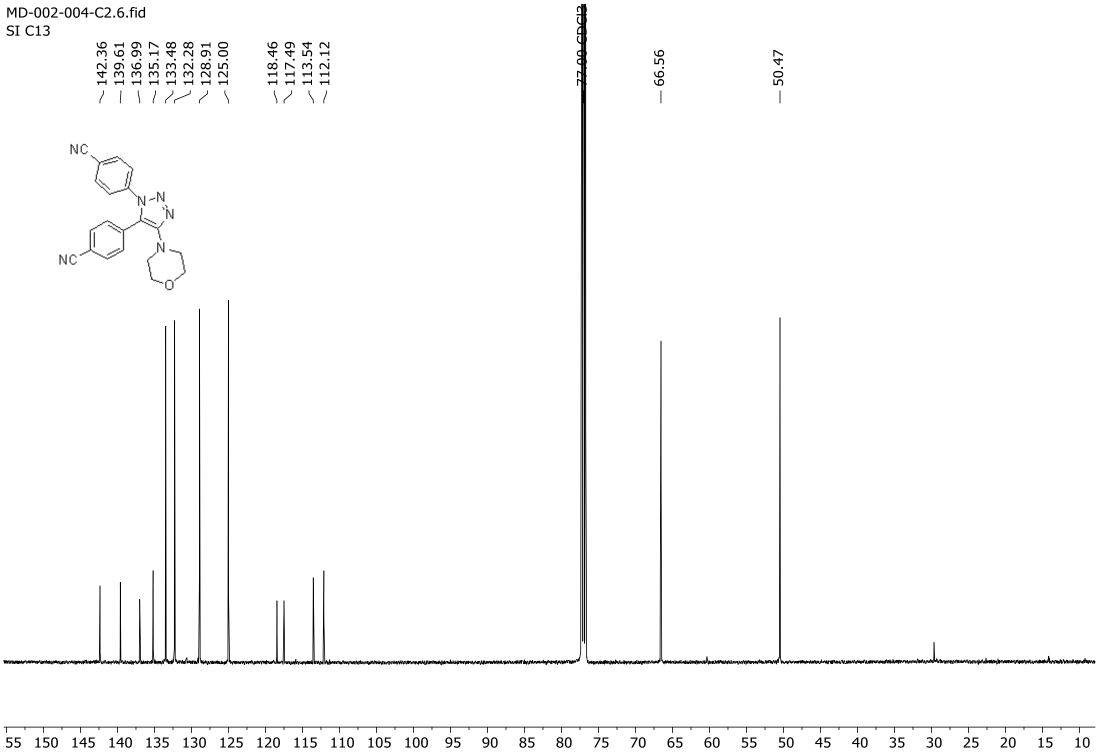

# $^{1}$H NMR of 3e

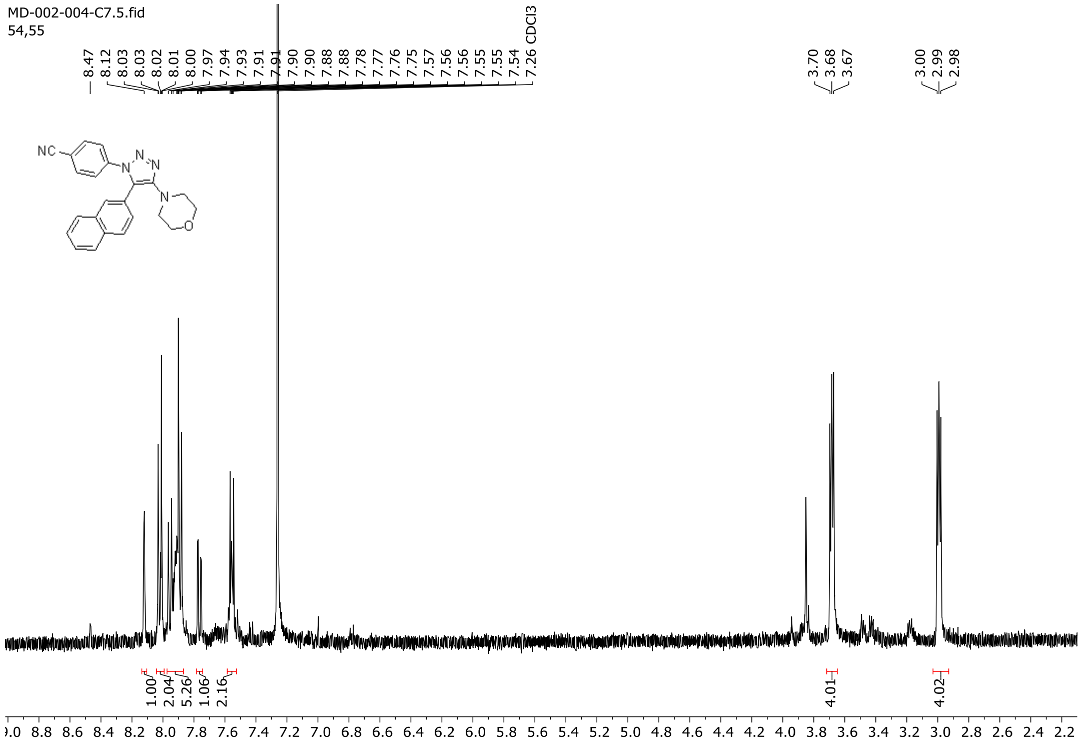

# $^{13}$C NMR of 3e

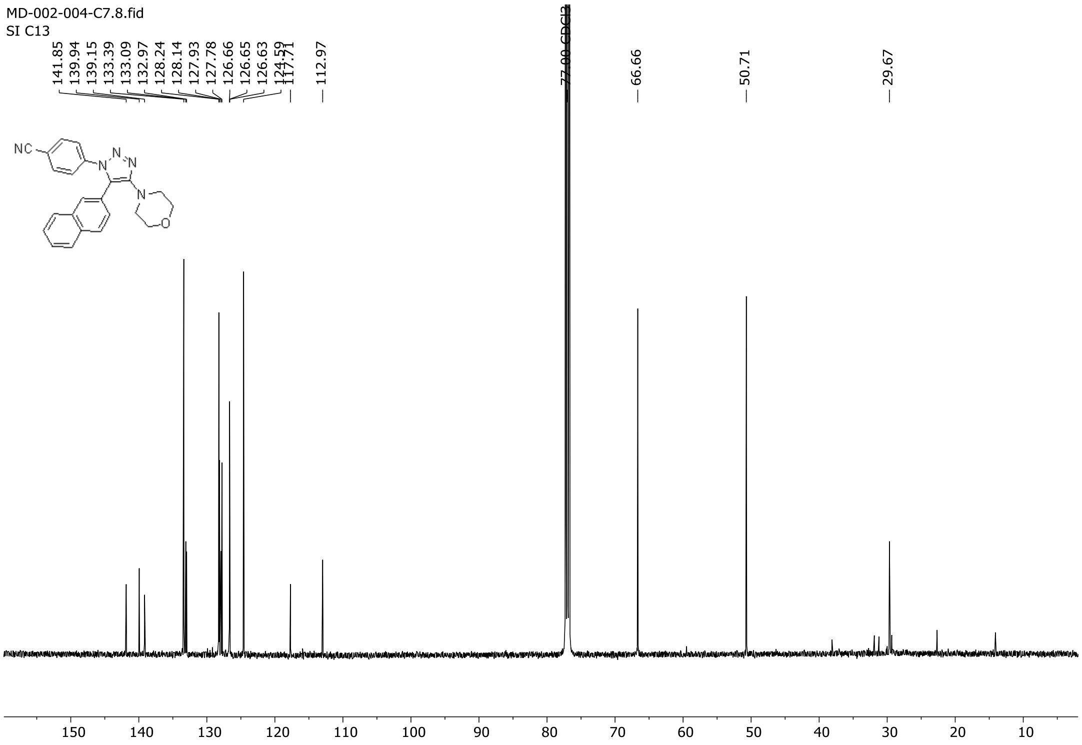

## $^{1}$H NMR of 4a

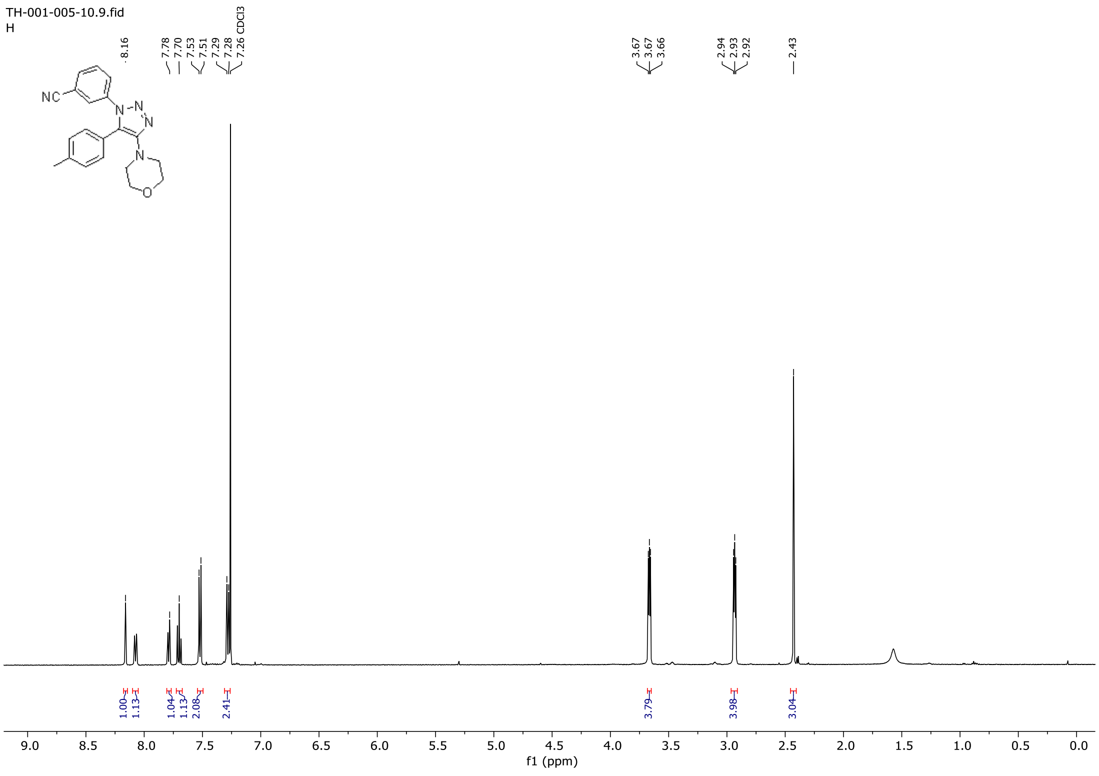


## $^{13}$C NMR of 4a

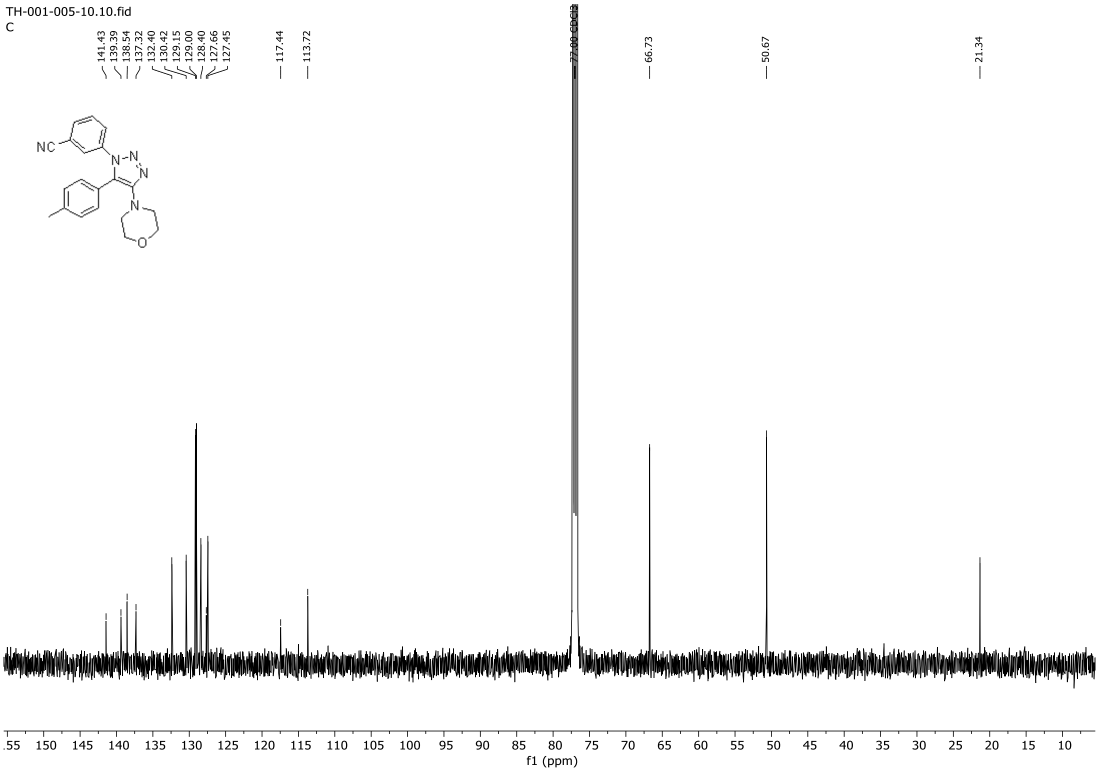

## $^{1}$H NMR of 4b

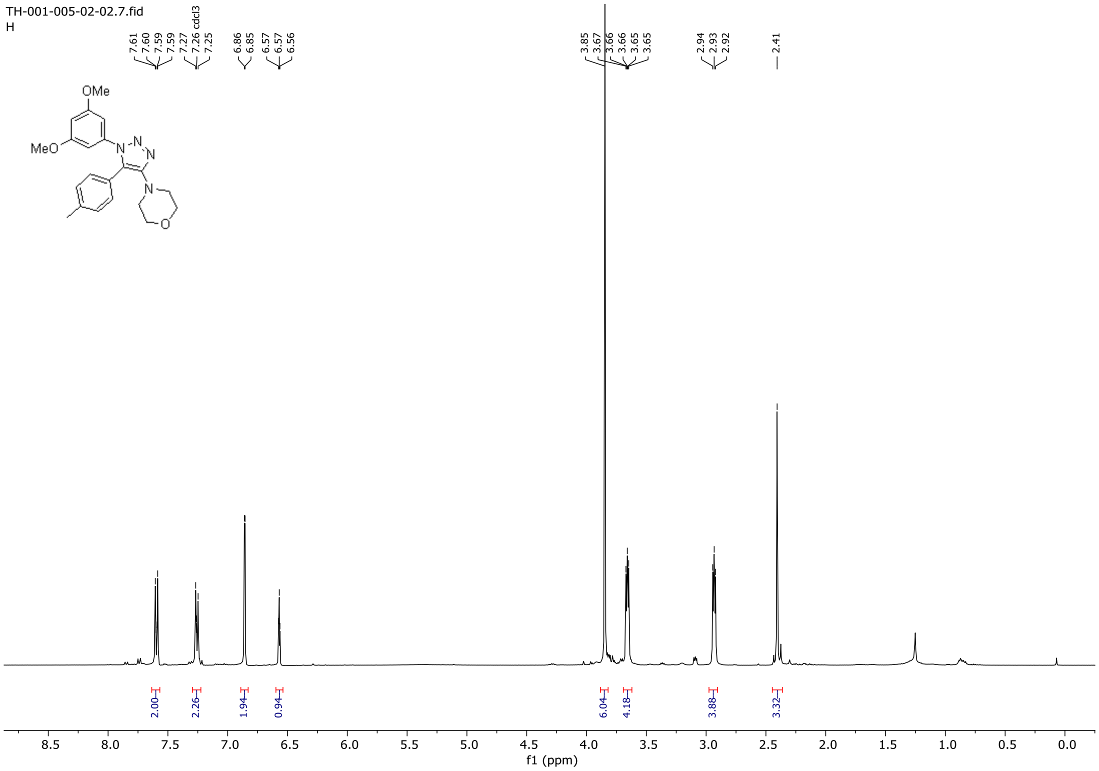


## $^{13}$C NMR of 4b

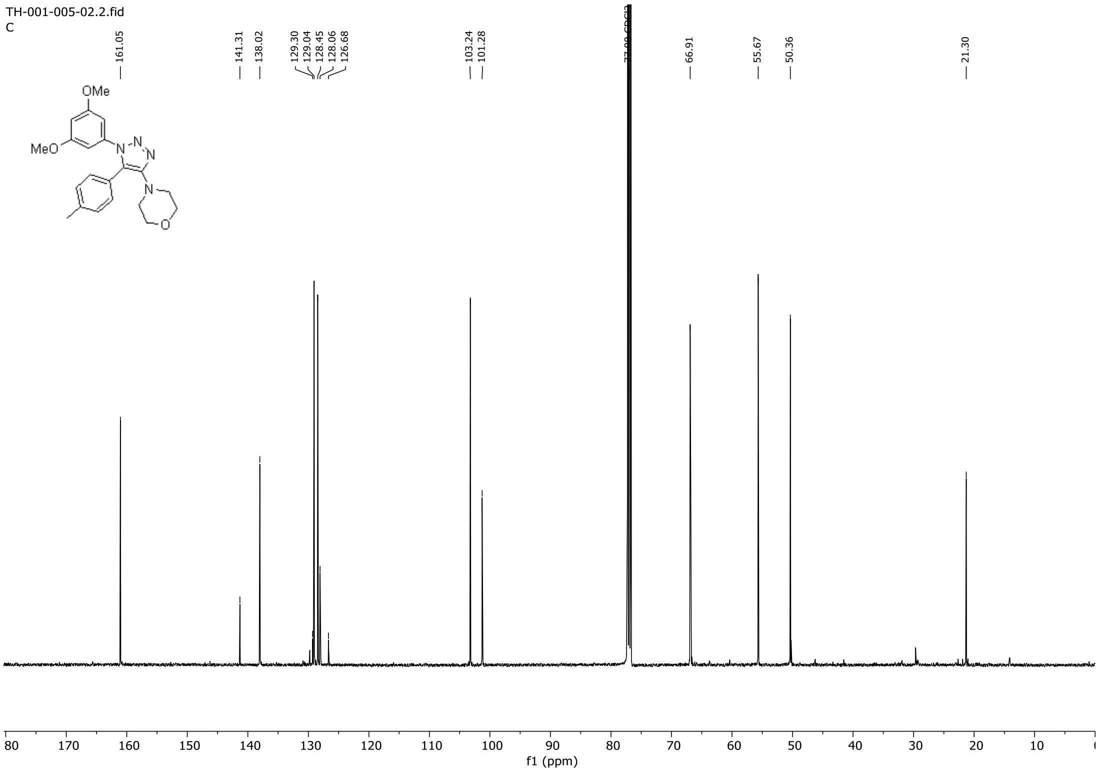

# $^{1}$H NMR of 4c

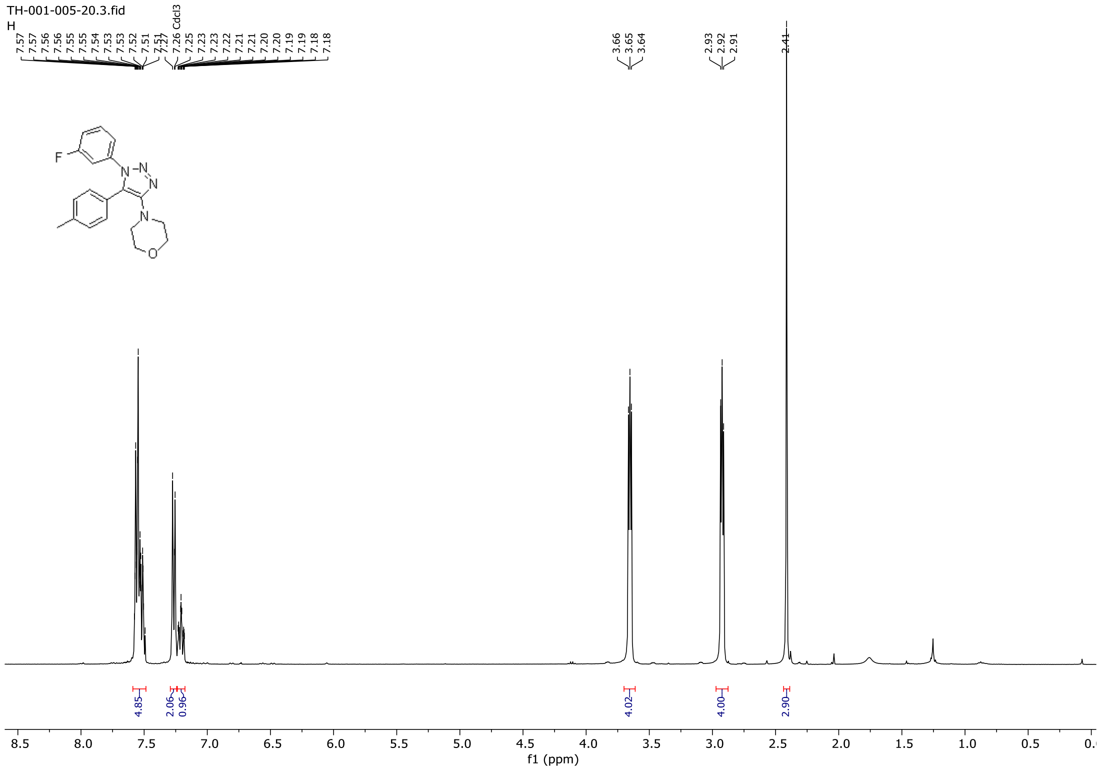


# $^{19}$F NMR of 4c

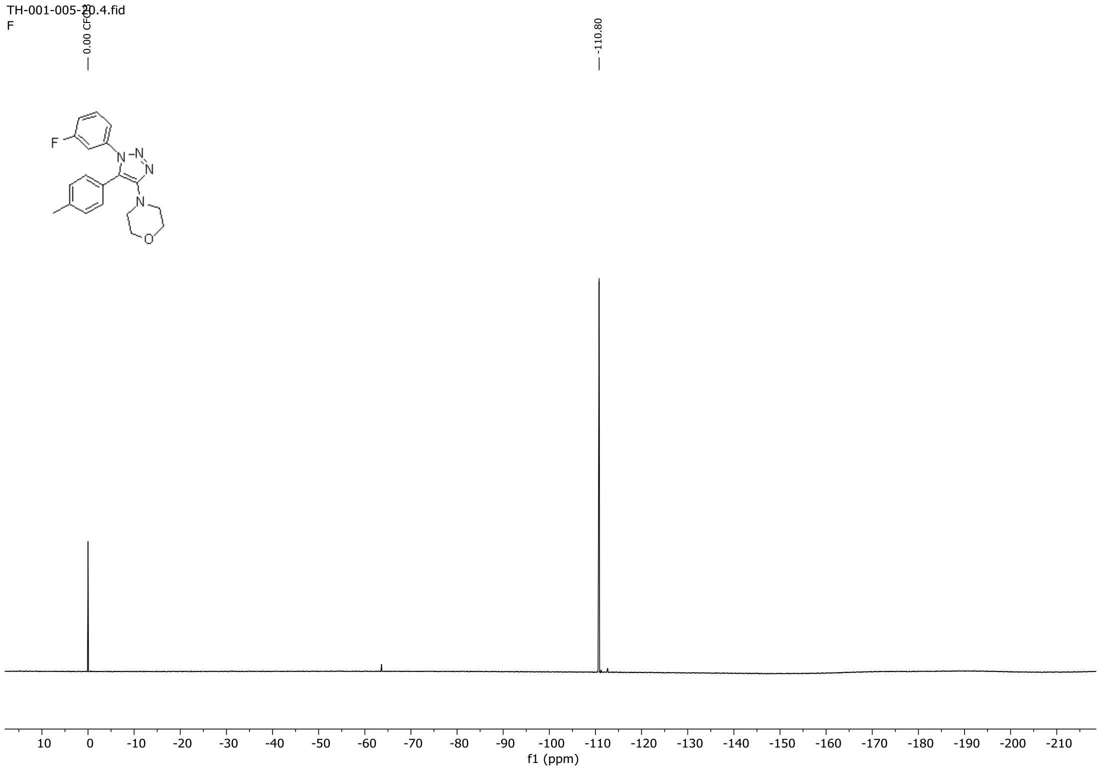

## $^{13}$C NMR of 4c

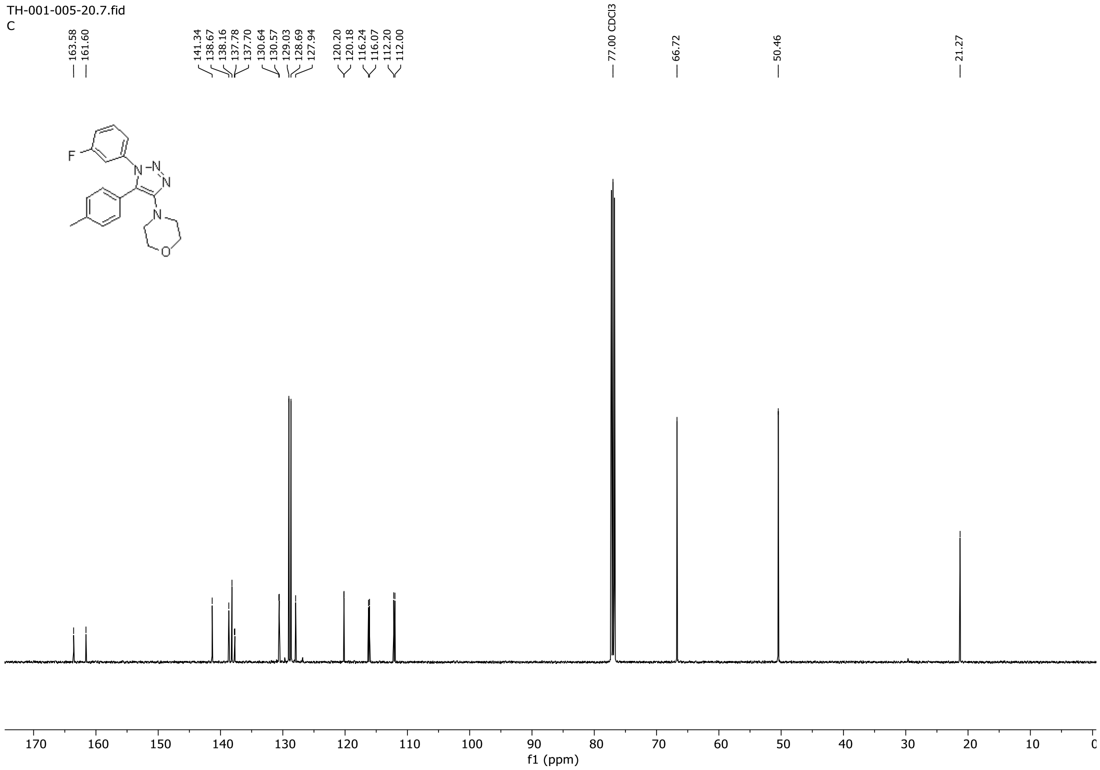


## $^{1}$H NMR of 4d

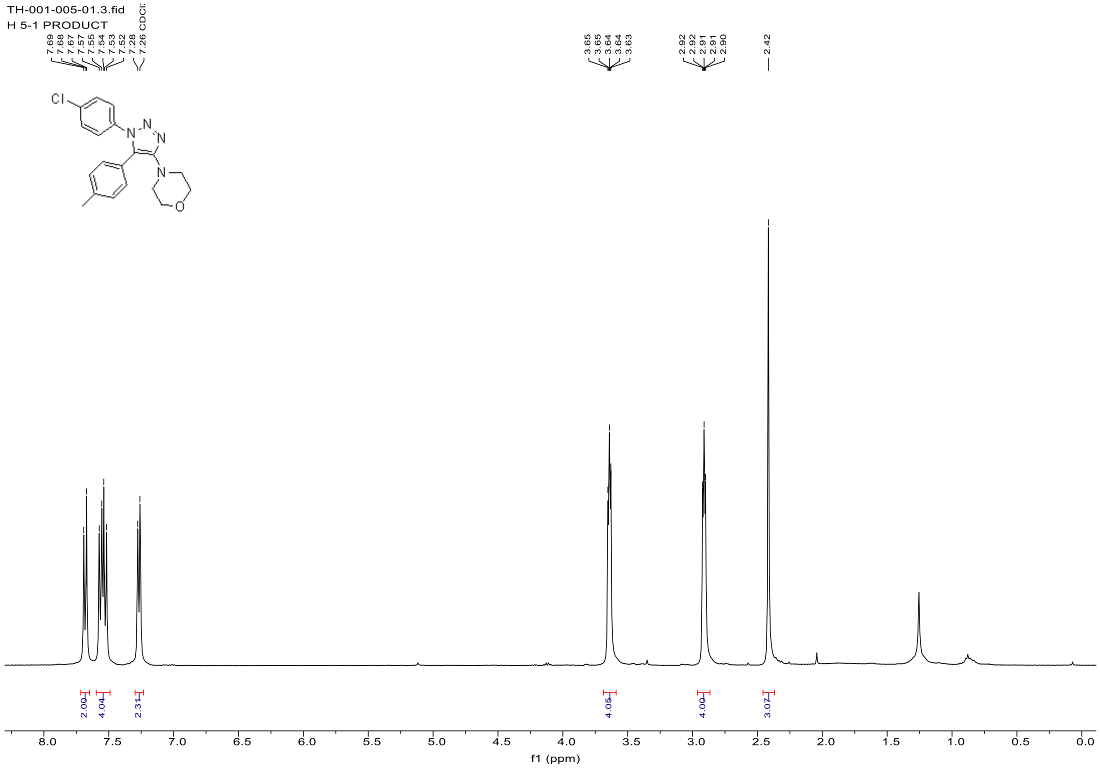

# $^{13}$C NMR of 4d

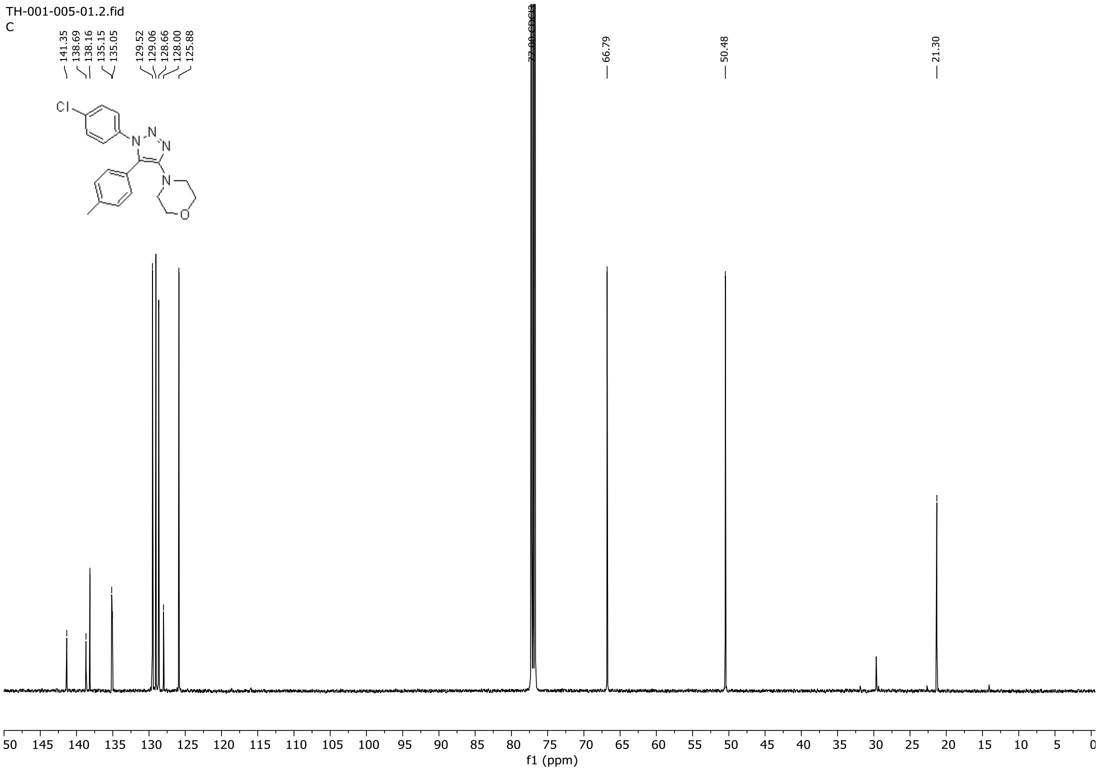


# $^{1}$H NMR of 4e

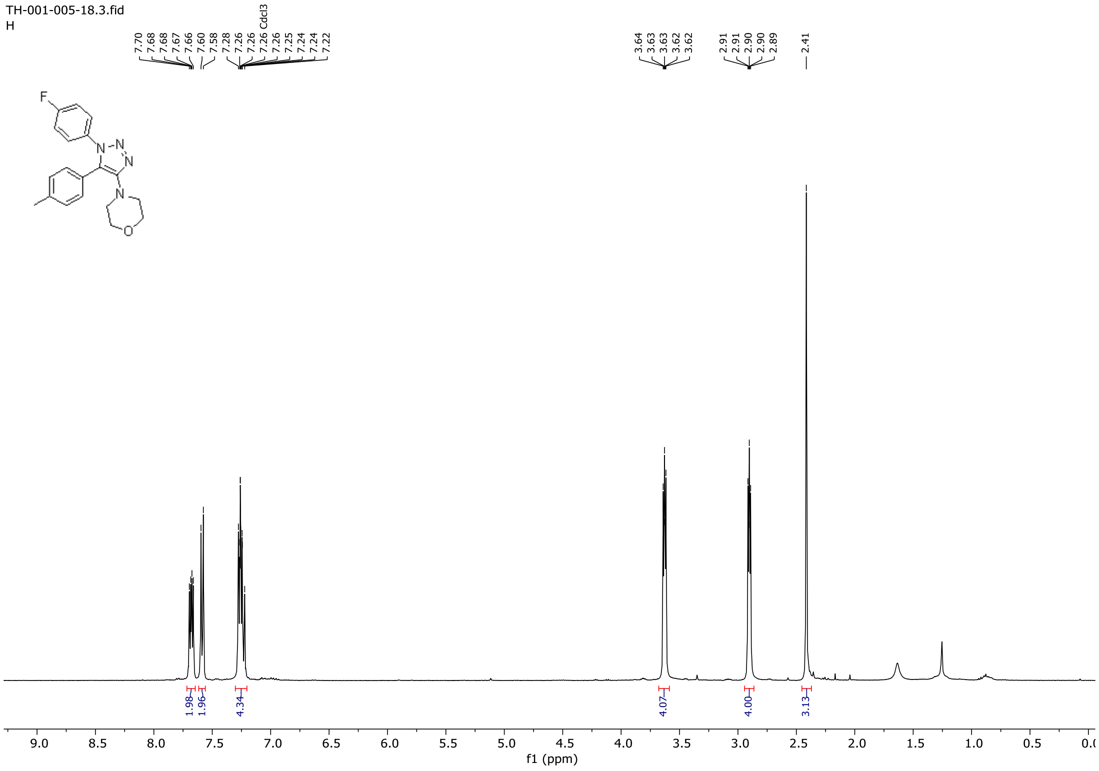

# $^{19}$F NMR of 4e

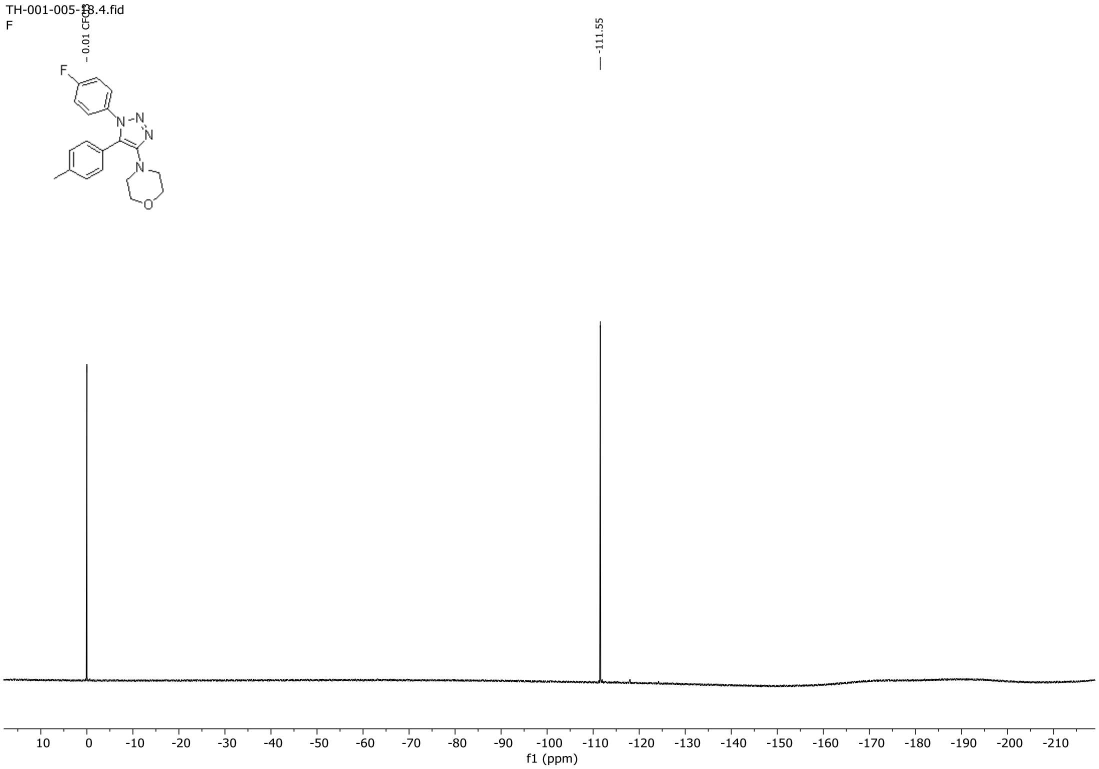


# $^{13}$C NMR of 4e

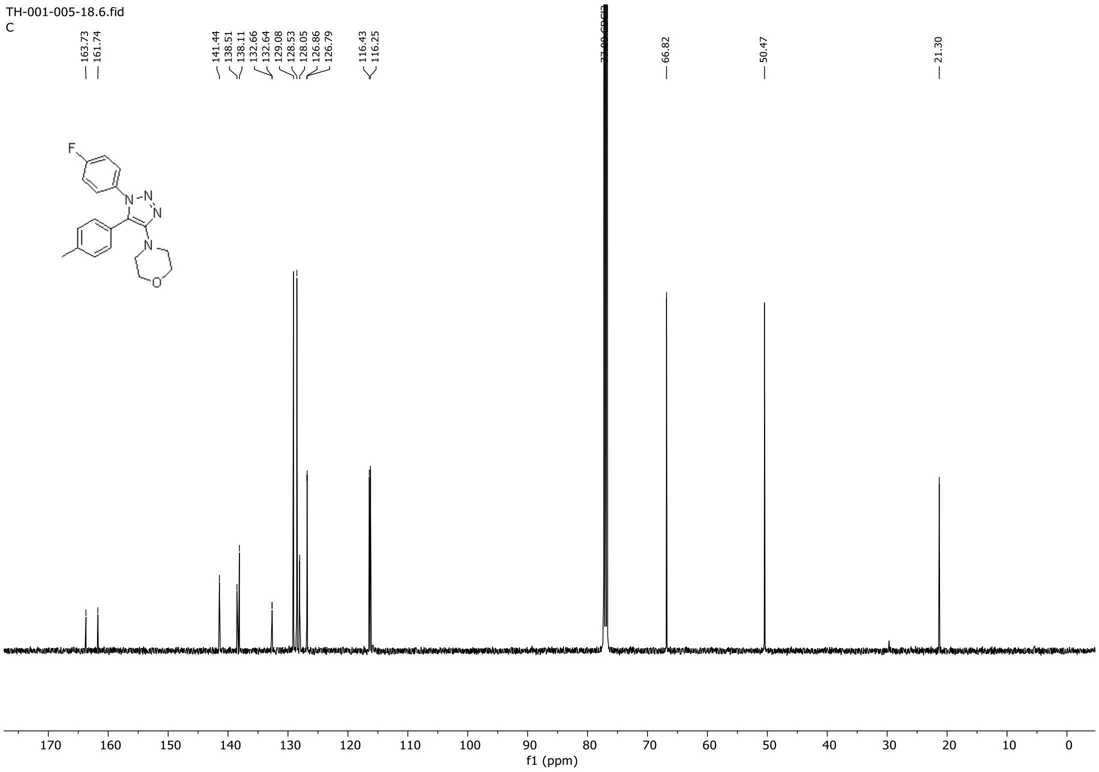

# $^1$H NMR of 4f

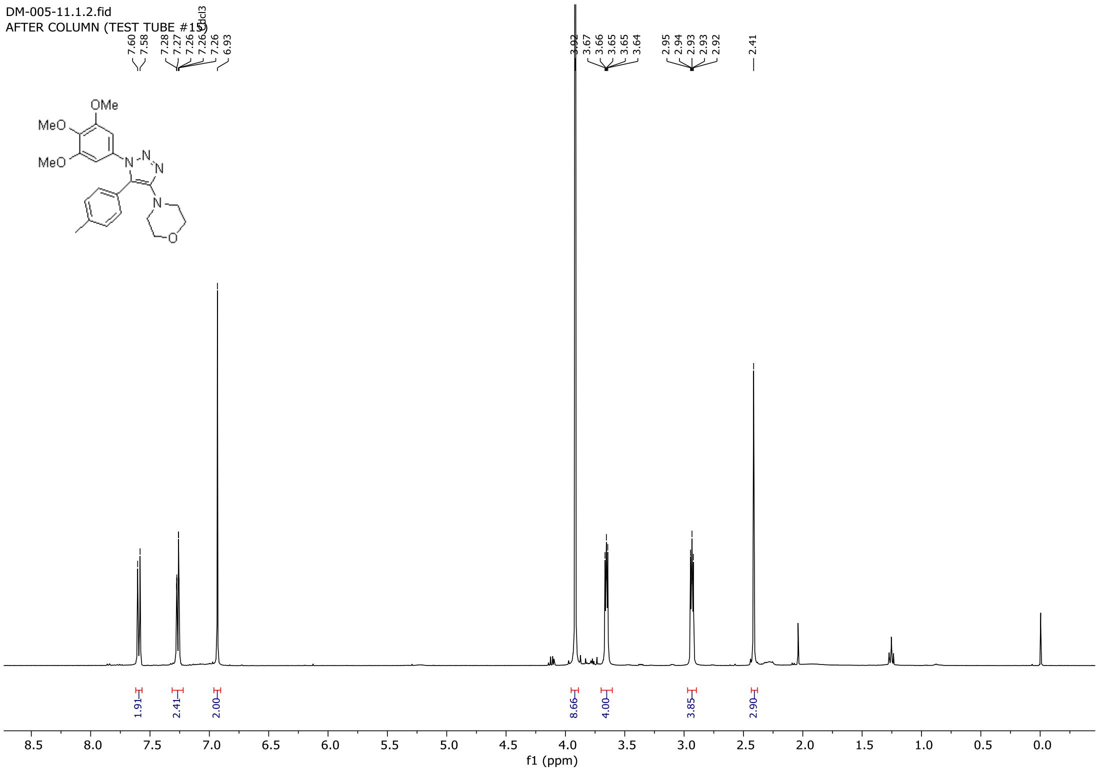


# $^{13}$C NMR of 4f

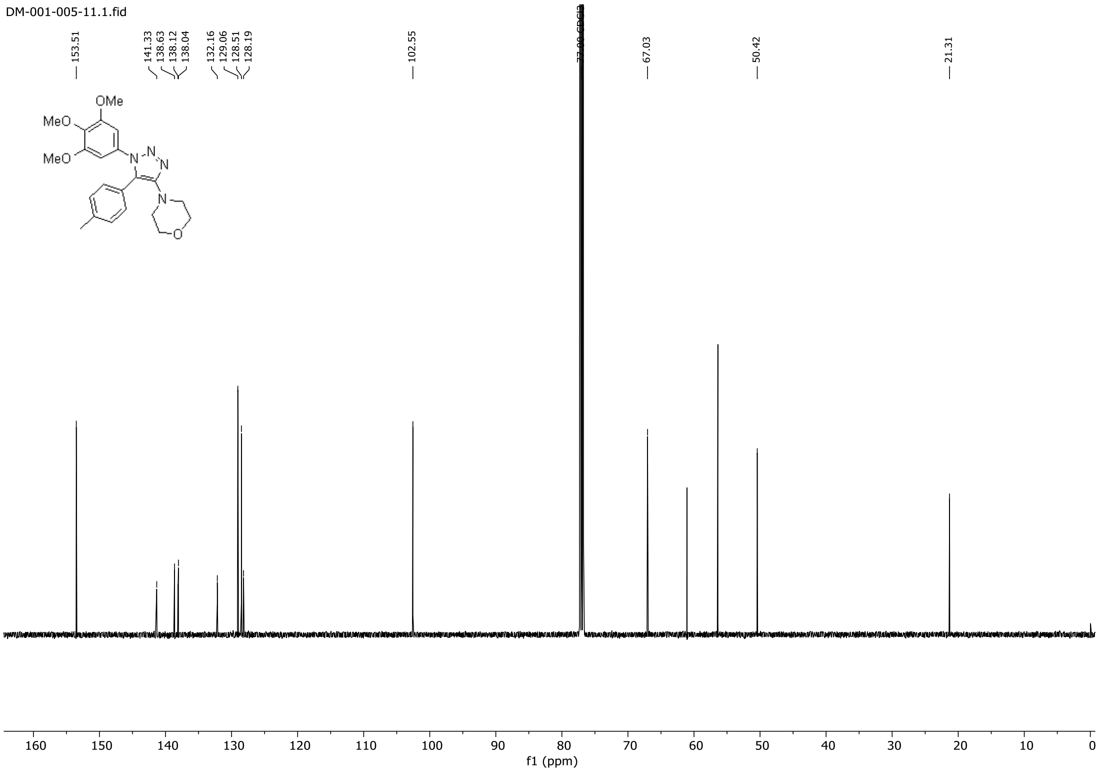

# $^{1}$H NMR of 4g

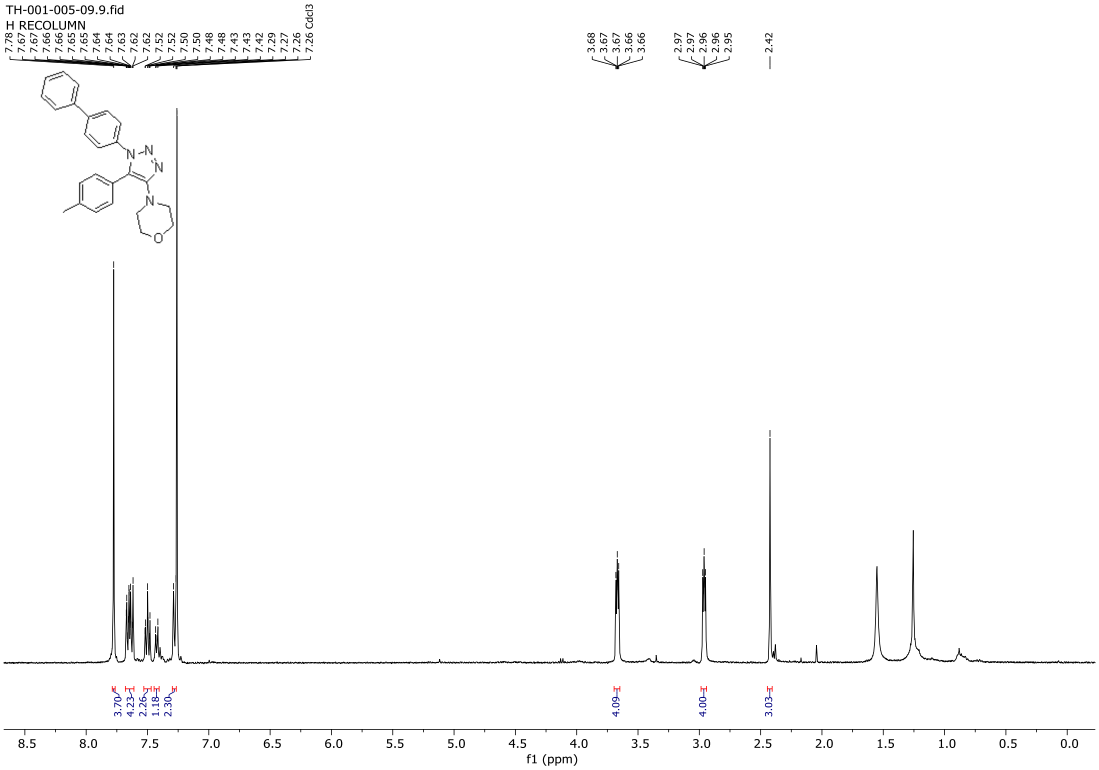


# $^{13}$C NMR of 4g

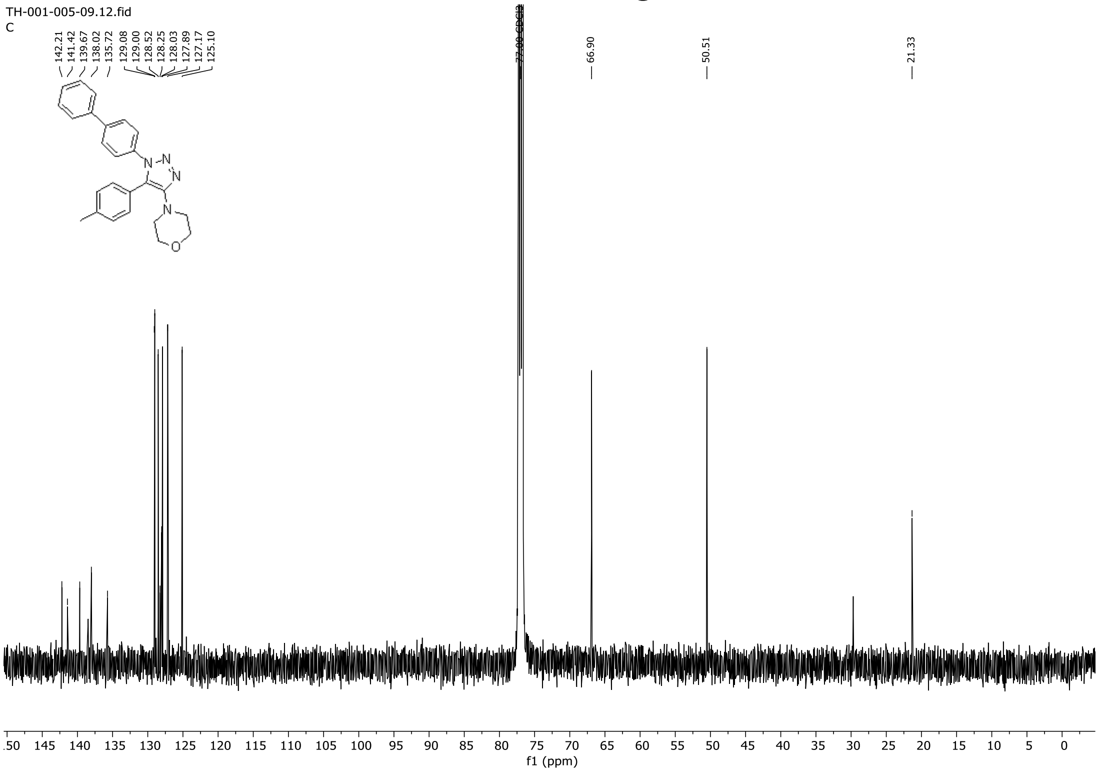

# $^1$H NMR of 4h

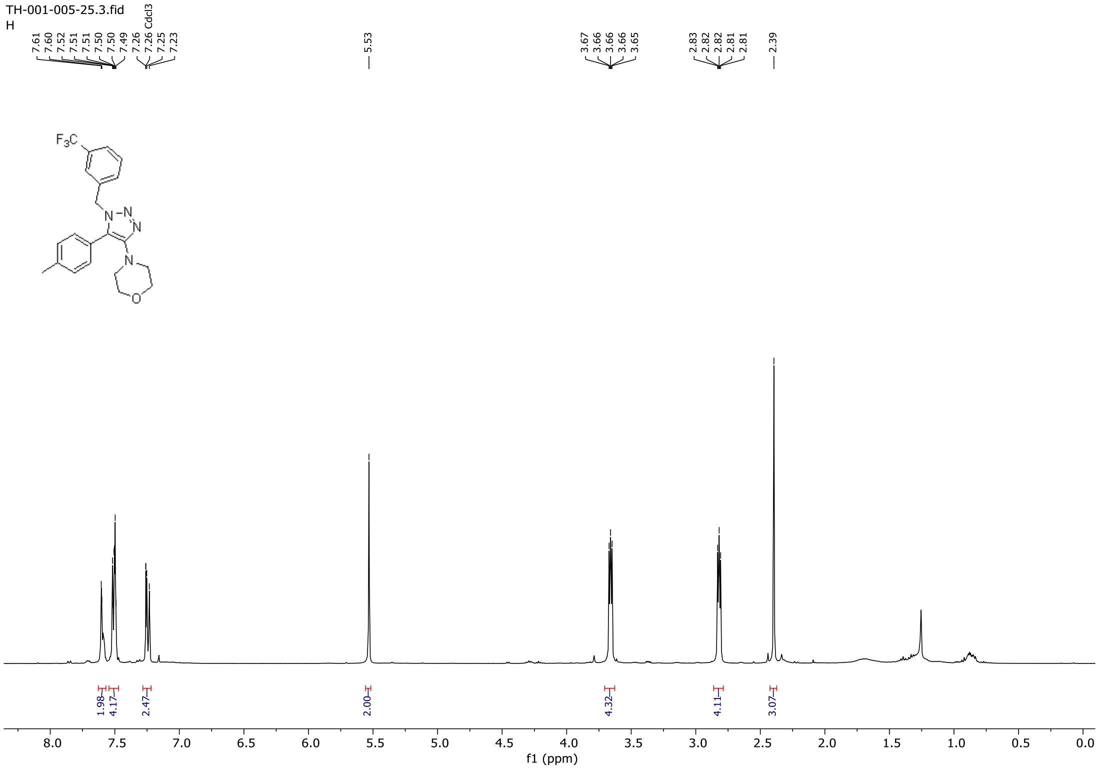


# $^{19}$F NMR of 4h

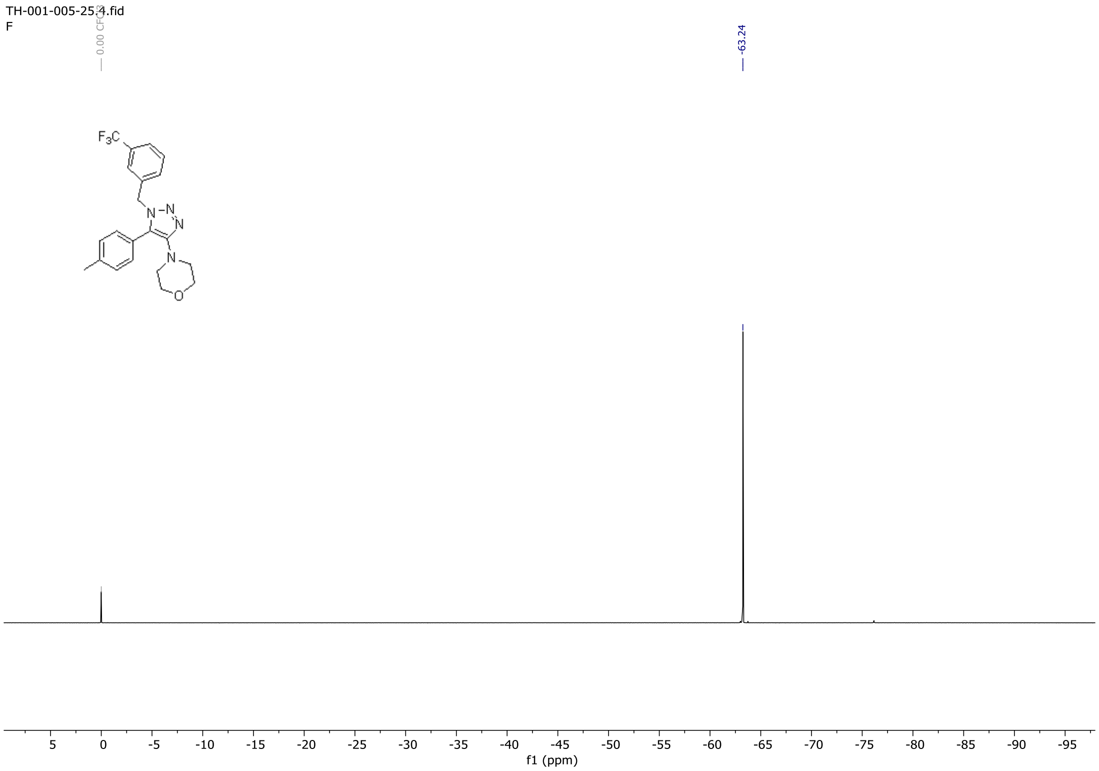

## $^{13}$C NMR of 4h

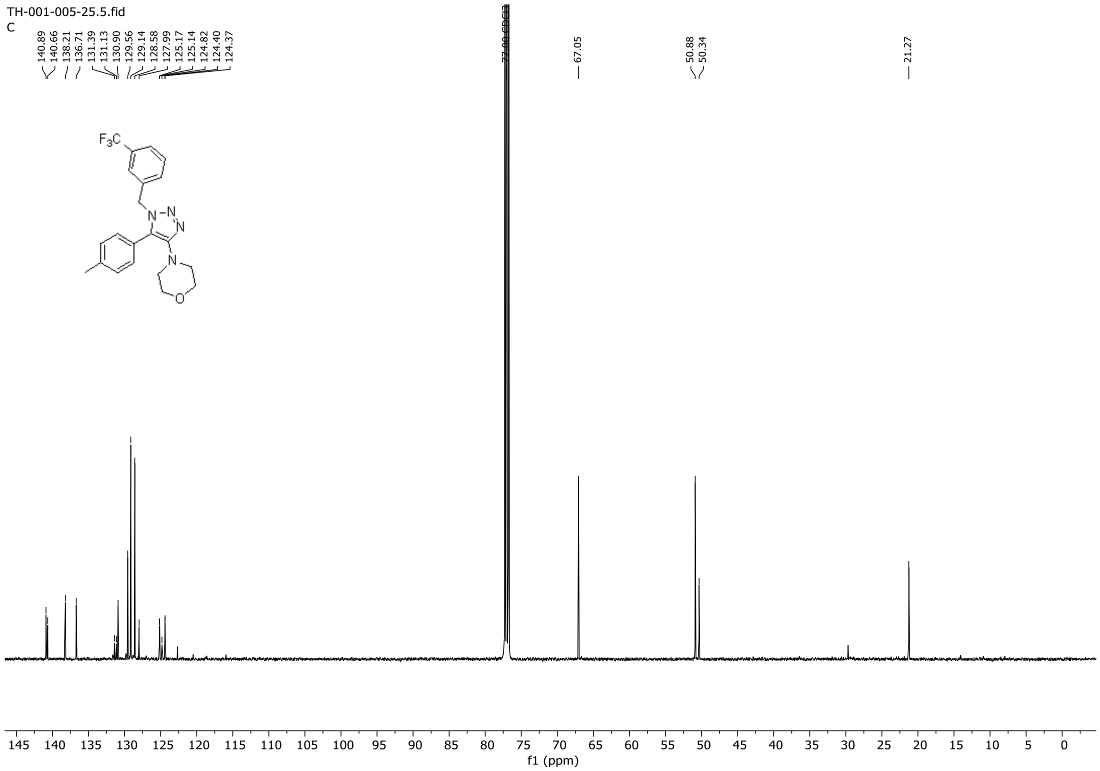


## $^{1}$H NMR of 5a

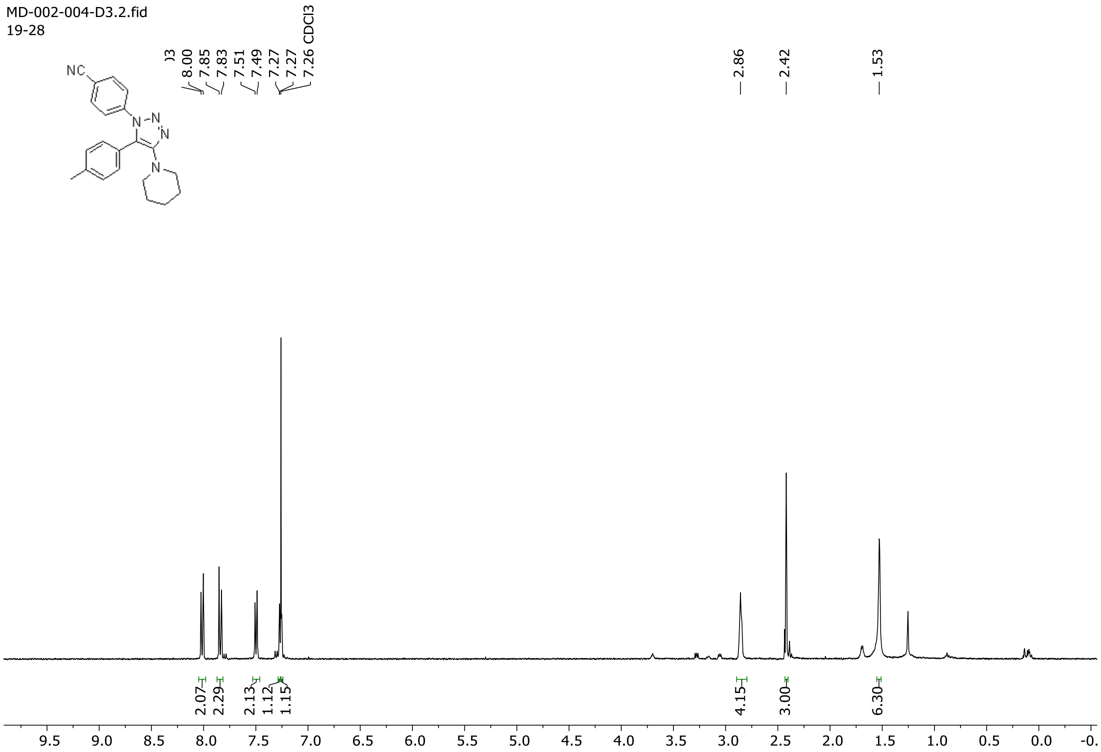

## $^{13}$C NMR of 5a

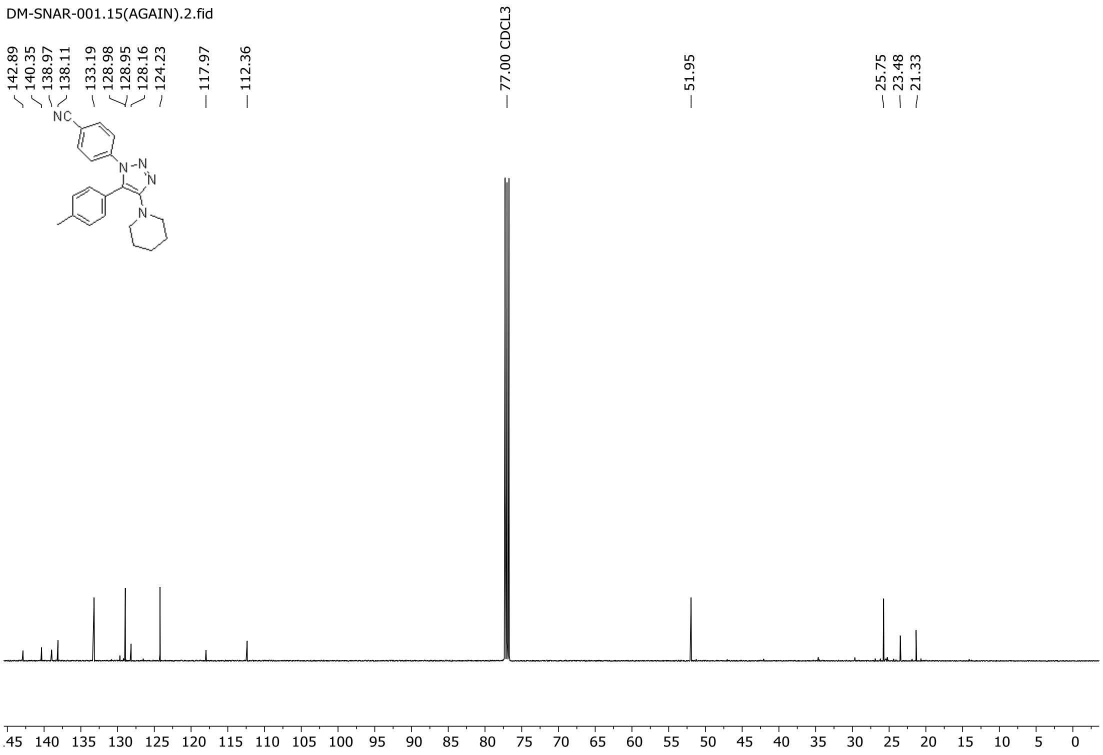


## $^{1}$H NMR of 5b

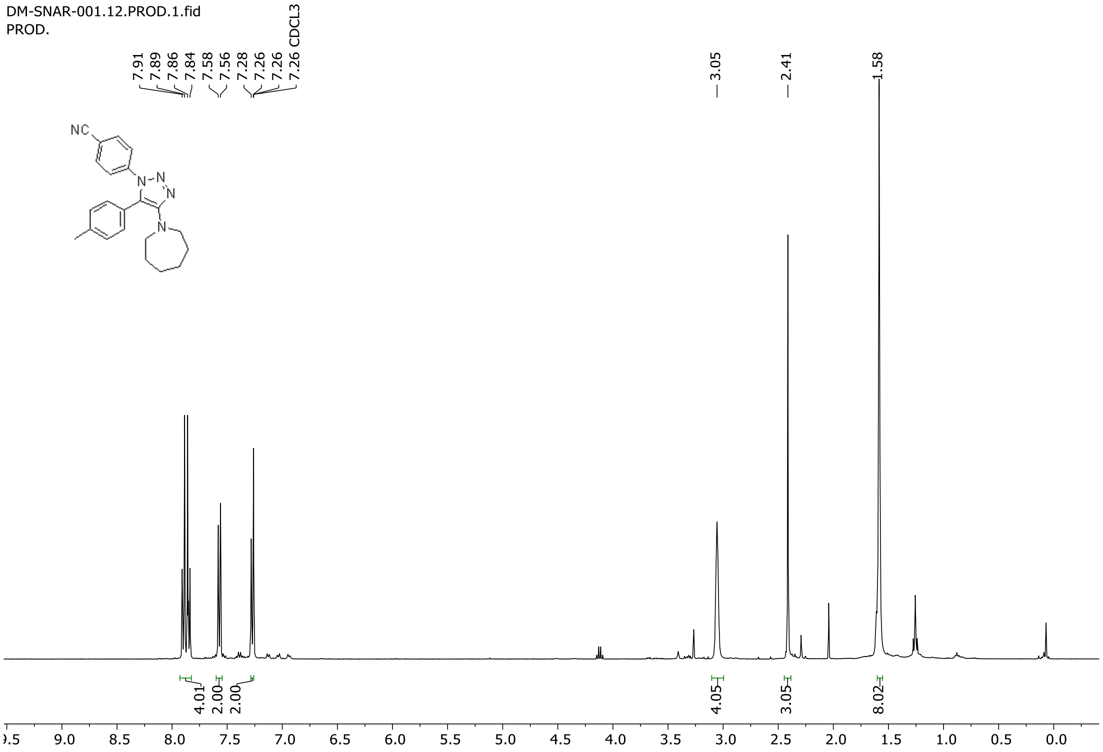

# $^{13}$C NMR of 5b

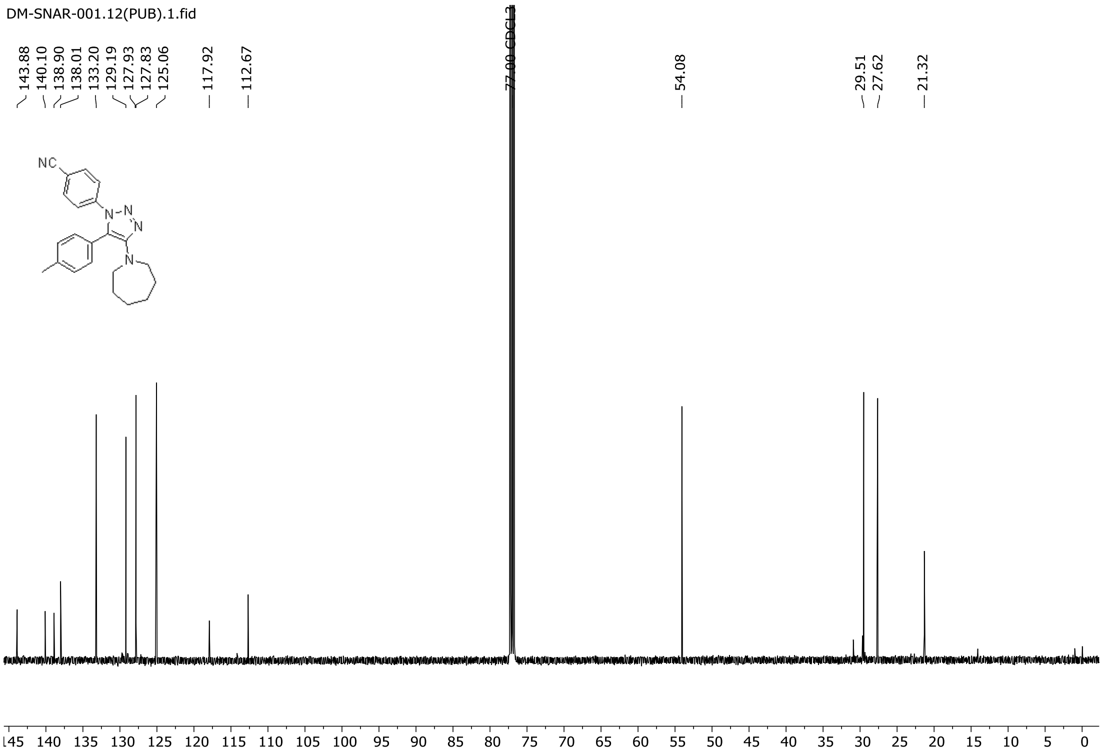

DM-SNAR-001.12(PUB).1.fid
143.88
140.10
138.90
138.01
133.20
129.19
127.93
127.83
125.06
117.92
112.67
77.00 CDCl3
54.08
29.51
27.62
21.32
NC
N
N
N
N
145 140 135 130 125 120 115 110 105 100 95 90 85 80 75 70 65 60 55 50 45 40 35 30 25 20 15 10 5 0

## 12. References:


1. D. R. Strobach, *The Journal of Organic Chemistry*, 1971, **36**, 1438-1440.
2. V. G. Nenajdenko, G. N. Varseev, V. N. Korotchenko, A. V. Shastin and E. S. Balenkova, *Journal of Fluorine Chemistry*, 2003, **124**, 115-118.
3. S. A. Fuqua, W. G. Duncan and R. M. Silverstein, *The Journal of Organic Chemistry*, 1965, **30**, 1027-1029.
4. L. Yu, M.-L. Tang, C.-M. Si, Z. Meng, Y. Liang, J. Han and X. Sun, *Organic Letters*, 2018, **20**, 4579-4583.
5. K. Barral, A. D. Moorhouse and J. E. Moses, *Organic Letters*, 2007, **9**, 1809-1811.
6. S. Oekchuae, J. Sirirak, P. Charoensuksai, P. Wongprayoon, N. Chuaypen, J. Boonsombat, S. Ruchirawat, P. Tangkijvanich, A. Suksamrarn and P. Limpachayaporn, *Pharmaceuticals*, 2022, **15**, 504.
7. F. Pagliai, T. Pirali, E. Del Grosso, R. Di Brisco, G. C. Tron, G. Sorba and A. A. Genazzani, *Journal of Medicinal Chemistry*, 2006, **49**, 467-470.
8. Z.-C. Dai, Y.-F. Chen, M. Zhang, S.-K. Li, T.-T. Yang, L. Shen, J.-X. Wang, S.-S. Qian, H.-L. Zhu and Y.-H. Ye, *Organic & Biomolecular Chemistry*, 2015, **13**, 477-486.
9. M. Payne, A. L. Bottomley, A. Och, H. G. Hiscocks, A. P. Asmara, E. J. Harry and A. T. Ung, *Bioorganic & Medicinal Chemistry*, 2022, **57**, 116648.
